# Single-cell atlas of human penile corpus cavernosum reveals cellular and functional heterogeneity of aging-related erectile dysfunction

**DOI:** 10.3389/fendo.2025.1671482

**Published:** 2025-10-29

**Authors:** Bailing Zhang, Xueheng Zhao, Jian Cao, Zhizhong Liu, Xiaoyan Wang, Ran Li, Hao Bo, Kongrong Xu, Jingtao Guo

**Affiliations:** ^1^ State Key Laboratory of Organ Regeneration and Reconstruction, Institute of Zoology, Chinese Academy of Sciences, Beijing, China; ^2^ University of Chinese Academy of Sciences, Beijing, China; ^3^ Beijing Institute for Stem Cell and Regenerative Medicine, Beijing, China; ^4^ NHC Key Laboratory of Human Stem Cell and Reproductive Engineering, Institute of Reproductive and Stem Cell Engineering, School of Basic Medical Science, Central South University, Changsha, China; ^5^ School of Nursing, Health Science Center, Hunan Normal University, Changsha, China; ^6^ Department of Urology, The Affiliated Cancer Hospital of Xiangya School of Medicine, Central South University/Hunan Cancer Hospital, Changsha, China; ^7^ Clinical Research Center for Reproduction and Genetics in Hunan Province, Reproductive and Genetic Hospital of CITIC-Xiangya, Changsha, China; ^8^ Center of Reproductive Medicine, Maternity and Child Health Care of Guangxi Zhuang Autonomous Region, Nanning, China

**Keywords:** aging-related ED, penile tissue, single-cell RNA sequencing, SMC, EC, Macro

## Abstract

**Background:**

With the acceleration of the aging process, the prevalence and severity of erectile dysfunction (ED) related to aging continue to rise, significantly impacting the physical and mental health of patients and their partners.

**Objectives:**

The objective of this study was to elucidate the cellular and transcriptomic heterogeneity and immune microenvironment of the aging penile corpus cavernosum in aging-related ED (ARED), while also exploring the potential pathological mechanisms driving ARED pathogenesis.

**Materials and methods:**

In this study, we performed single-cell RNA sequencing on penile tissues from six older patients with ARED, including four older mild ED (OmED) and two older severe ED (OsED) cases, and compared with three younger healthy controls from public data.

**Results:**

Through clustering and comparative analysis, we identified nine major cell types including immune cells. Our research revealed the heterogeneity of smooth muscle cells (SMC), endothelial cells (EC) and immune cells in penile environment, and determined the key cell subsets, such as C1_RERGL (a subcluster of SMC) and Macro_CXCL8, and molecular features involved in the pathogenesis of ARED. Furthermore, by constructing a comprehensive cellular communication network, the signals and interactions between macrophages (Macro) and other cell types were emphasized. We found that the interaction of ligand-receptor pairs such as CXCL2/3/8 – ACKR1 and HLA-E – KLRK1 were enhanced in the ARED group.

**Discussion and conclusion:**

The mild ED phase represents a critical window of cellular functional transition in penile tissue. In ARED, we observed functional dysfunction of EC and SMC, accompanied by changed expression levels of key genes. Concurrently, the pro-inflammatory Macro_CXCL8 exhibited increased proportion in the microenvironment, which may contribute to disease progression. This work not only provides a detailed description of the cellular atlas of the aging penis but also offers new insights into its role in the pathogenesis of ARED and may provide potential targets for the development of new therapies for ARED.

## 1 Introduction

Erectile dysfunction (ED), which has replaced the term impotence, is defined as the persistent inability to achieve or maintain an erection sufficient for satisfactory sexual performance (1). ED is one of the most prevalent chronic disorders, affecting 52% of men aged 40 to 70 years, with varying degrees of severity, and its prevalence increases with age (2). As global populations age, the worldwide prevalence of ED is projected to reach 322 million by 2025, posing a significant health concern that impacts the quality of life for both patients and their partners (3). Aging is an independent predictor of ED, regardless of other comorbidities (4). A study suggests that sexual desire does not decline significantly in aging men, who remain sexually active throughout their lives, while their partners experience a marked decline in desire with age (5). Furthermore, ED shares several risk factors and pathophysiological mechanisms with cardiovascular disease (CVD) and is considered a precursor and marker for future CVD events (6), providing a critical opportunity for diagnosis, prevention, and treatment of future cardiovascular issues.

Phosphodiesterase type 5 inhibitors (PDE5is), such as sildenafil, tadalafil, and vardenafil, are considered a first-line treatment for most men with ED by increasing levels of cyclic guanosine monophosphate (cGMP) (7). However, 30-40% of patients may not respond to this oral therapy, often due to underlying conditions like diabetes and severe neurological or vascular diseases, which can lead to significant vascular dysfunction and inadequate nitric oxide (NO) production, hindering the effectiveness of PDE5is in inducing penile erection (8). Men with compromised penile vasculature also do not benefit from PDE5i treatment (9). Common side effects associated with PDE5is include headache, facial flushing, and nasal congestion, which may be exacerbated in older individuals (10). Additionally, it is important to consider whether patients are using nitrates, as combining these medications can increase the risk of severe hypotension (11).

The morphological and physiological mechanisms underlying aging-related ED (ARED) involve oxidative stress, which leads to inflammation and cumulative damage over time (12). For instance, the age-related loss of functional corporal smooth muscle is primarily driven by oxidative stress, resulting in vasculogenic dysfunction such as arterial insufficiency and/or venous leakage, ultimately contributing to ED (13). However, the specific cellular composition and the molecular and functional changes in human penile tissue as it ages and progresses toward ED remain largely unexplored. Moreover, while many studies on the penile corpus cavernosum (CC) have been performed using rat models, there are significant structural and functional differences between human and rat corpora cavernosa, particularly regarding the spatial heterogeneity and species specificity of smooth muscle cells (14, 15). Thus, caution is warranted when attempting to directly apply findings from rat model studies to humans.

In this study, we collected penile tissues from six older men with ED and integrated public data from younger individuals with normal erectile function to conduct a single-cell resolution transcriptomic analysis. By constructing a comprehensive single-cell atlas that includes ARED patients, we characterized the heterogeneity of cell subsets and their molecular changes. This work provides valuable insights into the cellular microenvironment and potential pathological mechanisms underlying ARED.

## 2 Materials and methods

### 2.1 Sample collection and data acquisition

This research was approved by the Ethics Committee of the Hunan Cancer Hospital (no. KY2024100) (Changsha, China). Informed consent was obtained from all patients before surgery. The study includes six older patients with penile carcinoma, and non-tumoral penile tissues were obtained from the tumor margin during the resection surgery. The International Index of Erectile Function-5 (IIEF-5) was used to diagnose the presence and severity of ED. Based on their IIEF-5 scores, the six older patients with ED were categorized into two groups: older mild ED (OmED, scores 12-21) and older severe ED (OsED, scores 1-7). The clinical information for these samples is presented in **Supplementary Figure S1A**.

### 2.2 H&E and Masson staining

Fresh penile tissues were fixed in 4% paraformaldehyde for 12 h at 4 °C, by three times wash with 1 x PBS for 10 min each time. Then, the tissues were embedded using the conventional paraffin process and sectioned.

For H&E staining, penile tissue sections were baked in an oven (Yidi, China) at 65 °C for 1 h. Sections were deparaffinized with xylene, rehydrated with graded ethanol, and stained with hematoxylin (Beyotime, China) for 8 min at room temperature and rinsed under running water. Subsequent eosin staining (Beyotime, China) lasted 3-10 s, with thorough rinsing (~30 s) to ensure complete dye removal. The slides were rapidly passed through 70%, 80%, 95% and anhydrous ethanol in sequence, transparent in xylene 3 times for 2 min each time, and sealed with neutral resin (Sinopharm, China).

For Masson’s Trichrome Stain, the paraffin sections were dewaxed and stained with Weigert’s hematoxylin stain kit for 5-10 min. Then it was differentiated with an acidic ethanol differentiation solution for 5-15 s, returned to blue with Masson’s bluing buffer for 3-5 min, and then washed with distilled water for 1 min. Ponceau-acid fuchsin solution (Beyotime, C0189S, China) was used to stain muscle fibers to red for 5-10 min, followed by the distilled water for 5-10 s and the weak acid working solution for 1 min. Next, the collagen fibers were stained to blue with the brilliant green staining solution for 1 min, rinsed with distilled water, and performed with an acidic differentiation solution as well. The sections were dehydrated with 95% ethanol and anhydrous ethanol. Finally, they were dried with xylene three times and mounted with neutral balsam before observing under the microscope.

### 2.3 Tissue dissociation and single-cell isolation

After the surgical extraction, the fresh penile tissues were cut into pieces in the RPMI-1640 medium (Invitrogen) with 1% Penicillin/Streptomycin, and then enzymatically digested at 37 °C with agitation for 30 min using MACS *Tumor Dissociation Kit_mouse* (Miltenyi Biotec). Following dissociation, the cells were filtered through a 70-um and 40-um cell-strainer (BD) and centrifuged at 300g for 10 min. The resulting cell pellet was resuspended in red blood cell lysis buffer (Thermo Fisher) and incubated on ice for 2 min to lyse red blood cells. After two washes with PBS (Invitrogen), the cells were resuspended in PBS (containing 0.04% BSA) in preparation for single-cell RNA library construction.

### 2.4 Single-cell RNA-seq library construction and sequencing

The mRNA capture was performed on DNBelab C4 device (MGI). cDNA amplification and library construction were completed using the MGI DNBelab C series reagent Kit (MGI, 940-000047-00) according to the manufacturer’s protocol. All libraries were further sequenced by the DIPSEQ T1 sequencing platform after qualification by Qubit ssDNA Assay Kit (Thermo Fisher Scientific) and Agilent Bioanalyzer 2100. The raw sequencing data filtered and gene expression matrix was obtained using DNBelab C Series scRNA-analysis-software, in which processed reads were aligned to GRCh38 genome reference using STAR (v2.5.3).

### 2.5 Preprocessing and quality control of scRNA-seq data

Downstream analysis for scRNA-seq data was implemented by R package Seurat (v4.3.0) (16) (https://satijalab.org/seurat/). We first quantified the total number of captured cells for each sample (**Supplementary Figure S1A**) and then performed quality control. Cells were retained with 500-7000 number of expressed gene, 0-40,000 detected UMIs, and < 20% proportion of mitochondrial genes (< 15% for the dataset GSE206528).

Epithelial contaminated clusters were identified by the marker *EPCAM* and removed before integrated and further analyses. Doublets were identified and removed by R package DoubletFinder v2.0.3 (17) in each individual dataset. Retained the genes shared between the external dataset and this work.

### 2.6 Sample integration and batch effect correction

Given that our data and the external control (10× Genomics) originated from different platforms, we used Seurat to integrate them and eliminate the effect of batch effects. Specifically, we followed the standard workflow by applying the *FindIntegrationAnchors* and *IntegrateData* function with default parameters.

### 2.7 Unsupervised clustering analysis and cell type identification

The merged data was normalized by the NormalizeData function (normalization.method = “LogNormalize”) and scaled by the *ScaleData* function, while regressing out the percentage of mitochondrial genes, and then underwent principal component analysis (PCA) using the *RunPCA* function. The *ElbowPlot* function was used to determine the appropriated PCA dimensions. We applied the first 20 principal components (PCs) to construct shared nearest neighbor graph and performed dimensionality reduction with *RunUMAP* function. All cell clustering analysis was performed with *FindClusters* function at a resolution of 1.2 and annotated each cluster based on canonical marker genes. We identified nine major cell types, including four stromal cell types, four immune cell types and one neuroglial cell type. Major cell types and selected marker gene listed below: fibroblasts (FB: *LUM*, *FBLN1*, *COL1A1*, *PDGFRA*), endothelial cells (EC: *VWF*, *PECAM1*, *PODXL*), smooth muscle cells (SMC: *ACTA2*, *MYH11*, *ACTG2*), pericytes (PC: *PDGFRB*, *STEAP4*, *ABCC9*, *NOTCH3*), T cells/Natural killer cells (T/NK: *CD3D*, *CD3E*, *NKG7*, *GNLY*), monocytes/macrophages (Mono/Mac: *CD163*, *CD14*, *CD68*, *CD1C*, *CLEC10A*), mast cells (MC: *TPSB2*, *TPSAB1*, *MS4A2*), plasma cells (Plasma: *CD79A*, *MZB1*, *IGHA1*), and Schwann cells (SWC: *CDH19*, *S100B*, *MPZ*). Marker genes of each cell type were visualized via *FeaturePlot* and *DotPlot*. To identify cellular subsets within these cell types, we extracted them and performed re-clustering analysis.

### 2.8 Identification of differentially expressed genes and enrichment analysis

Differentially expressed genes (DEGs) for each cell type or subcluster were identified by the *FindAllmarkers* function *(*test.use=wilcox; min.pct=0.25; logfc.threshold=0.25). Briefly, DEGs for a given cell type were identified by comparing cells of that type to all other cell types using above parameters, and only the genes with a maximum adjust P value of 0.05 were considered as DEGs.

Cell type identity genes were defined as those exhibiting avg_log2FC > 1 (indicating upregulated expression) and p_val_adj < 0.05 relative to all other cell types, thereby conferring enhanced cell type specificity. We scored these cell types using the “AddModuleScore” function.

Pairwise differential gene expression analysis between given cell types or subclusters were identified by the *FindMarkers* function (Wilcoxon rank sum test) in Seurat.

All enrichment analysis on DEGs was performed using Metascape and visualized with R package ggplot2. Representative terms or pathways (p < 0.01) were displayed.

### 2.9 Gene set enrichment analysis

Enriched gene sets between two groups were identified using GSEA function from R package clusterProfiler (v4.10.1) (18). Hallmark gene sets were selected and downloaded from human Molecular Signatures Database (MSigDB) (19). Gene sets with adjust p-value < 0.05 were considered significant enriched gene sets. The results were visualized using the gseaNb function from the GseaVis package (https://github.com/junjunlab/GseaVis) and ridgeplot function from ggridges package.

### 2.10 Gene set variation analysis

To assess the potential changes in pathway activity across groups, we adopted Gene set variation analysis (GSVA) (20). GSVA was performed using the R package GSVA (v 1.51.17). The latest KEGG pathway gene sets was download using download_KEGG function in clusterProfiler (v 4.10.1). Representative pathways were displayed.

### 2.11 TF enrichment analysis

TF and regulon enrichment analysis were analyzed by pySCENIC (v 0.12.0) (21) with default parameters. TFs of hg38 was downloaded from Rcis Target (https://resources.aertslab.org/cistarget/tf_lists/). The output loom file was further analyzed with the R package ScopeLoomR (v0.13.0). The subcluster specific regulons were identified by calculating regulon specificity scores between subclusters of interest. The identified TF and its target genes were visualized by Cytoscape (v3.10.0) (22).

### 2.12 Cell-cell communication analysis

CellChat (v2.1.2) (23) package was used to predict the ligand-receptor communications between cell types and identify altered signaling. Normalized single cell data was used as input for analysis. The database CellChatDB.human, including “Secreted Signaling”, “ECM-Receptor” and “Cell-Cell Contact” ligand-receptor pairs, was selected to analyze the cell-cell communication. Comparison analysis of multiple datasets with same cell population compositions was performed according to the tutorial (https://github.com/jinworks/CellChat)

### 2.13 PROGENy analysis

The R package progeny (v1.24.0) was utilized to infer the activity of 14 signaling pathways in EC from gene expression. The top 1,000 target genes for generating the model matrix were used (24).

### 2.14 Single-cell metabolic analysis

The scMetabolism (v0.2.1) R package was used to quantify and visualize the metabolic features of each EC subclusters. VISION method and Kyoto Encyclopedia of Genes and Genomes (KEGG) pathways were used to quantify the metabolic activity. We filtered the metabolic pathways and the outcome were depicted using the DotPlot.metabolism function.

### 2.15 Gene set score analysis

The *AddModuleScore* function in Seurat was used to calculate module scores.

SenMayo (25) gene set and immune-associated Hallmark gene sets and human Natural killer cell mediated cytotoxicity gene set were downloaded from MSigDB database. Core ECM components were acquired from https://sites.google.com/uic.edu/matrisome/home, the M1, M2, Angiogenesis and phagocytosis gene sets were listed in **Supplementary Table S5**.

### 2.16 Statistical analysis

The staining data was statistically analyzed by GraphPad Prism (V8) with a one-way ANOVA test, results were presented as mean ± SD. Other data statistical analyses were done using R 4.3.2 including Wilcoxon test as described in the Figure legends. p.adj lower than 0.05 are considered statistically significant. *, **, ***, **** indicate p.adj <0.05, p.adj < 0.01, p.adj < 0.001 and p.adj < 0.0001 respectively.

## 3 Results

### 3.1 Global cellular landscape of human CC aging

**Figure 1:**
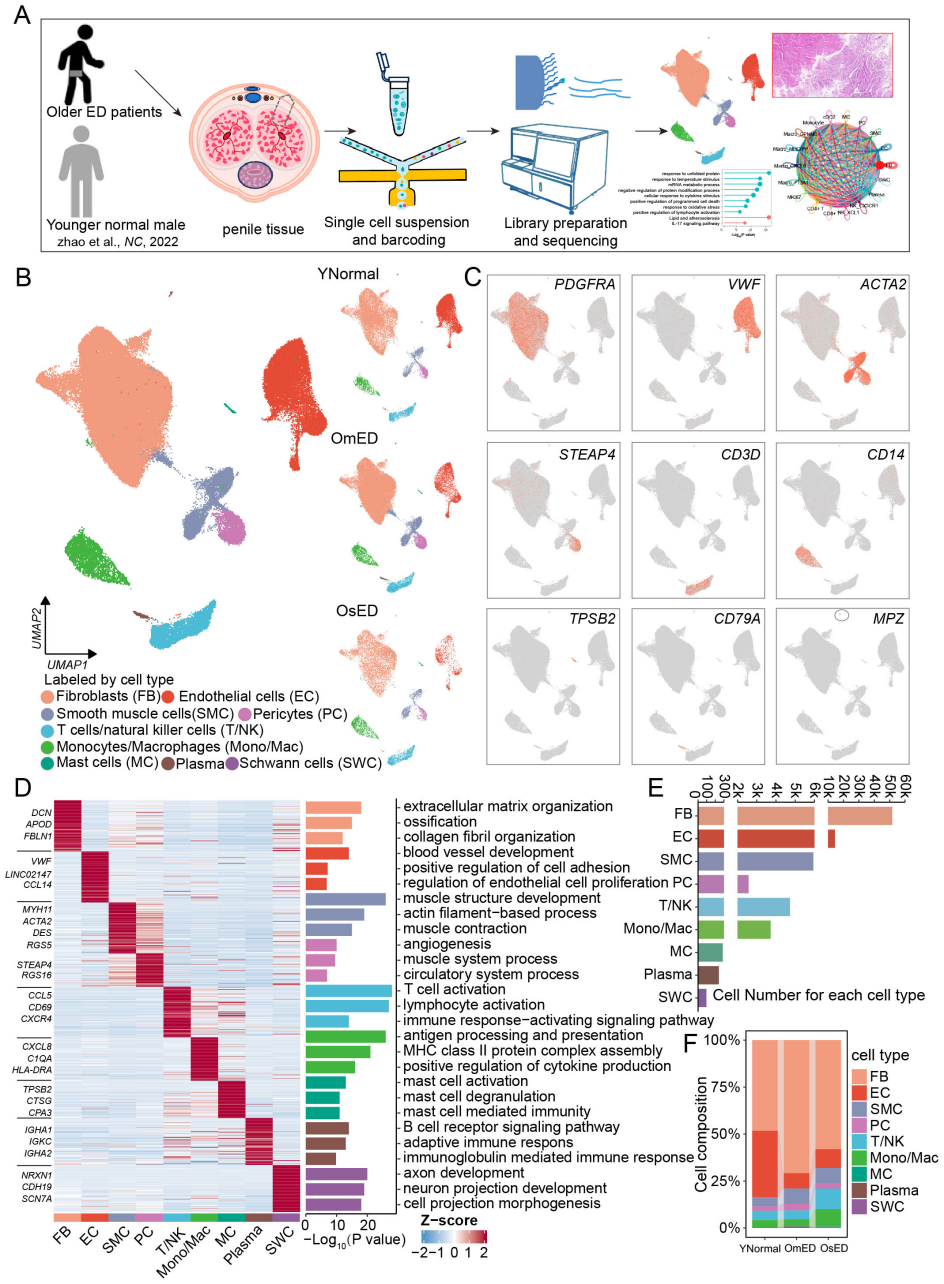
Global single-cell landscape for human CC in aging-related ED. **(A)** Schematic graph describing the experimental workflow. Human penile tissues from six older patients with erectile dysfunction were collected for single-cell RNA-seq. **(B)** Uniform manifold approximation and projection (UMAP) plots showing 83,743 cells from nine individuals (three younger normal males from GSE206528, four older mild ED patients and two older severe ED patients) separated into nine major cell types (left) and cell type distribution in YNormal, OmED and OsED groups (right). **(C)** Feature plots showing the expression distribution of marker genes for each cell type: *PDGFRA* (FB), *VWF* (EC), *ACTA2* (SMC), *STEAP4* (PC), *CD3D* (T/NK), *CD14* (Mono/Mac), *TPSB2* (MC), *CD79A* (Plasma) and *MPZ* (SWC). **(D)** Heatmap showing row z-score expression signatures of top 100 cell type specific genes. Top three marker genes for each cell type are shown on the left of heatmap (left). Enriched representative Gene Ontology (GO) terms for marker genes (right). **(E)** Bar plots showing the numbers of each cell type in human penile tissues. **(F)** Cell proportion of each cell type in different groups.

To investigate the cellular diversity and molecular characteristics of ARED, we collected penile tissues from six older patients with ED for single-cell RNA sequencing (scRNA-seq). This included four patients with mild ED (OmED) and two with severe ED (OsED), alongside three samples with normal erectile function from the dataset GSE206528 (26) (**Figure 1A**; **Supplementary Figure S1A**). Histological images from the three groups, stained with H&E and Masson’s trichrome stain, were presented in **Supplementary Figure S1B**. The aged penises with ED were characterized by a reduction in smooth muscle cells and an increase in collagen compared to younger penises with normal erections.

After stringent quality control and filtering, including the removal of doublets, we obtained 83,743 cells for downstream analysis (**Supplementary Figure S1C**). We utilized canonical integrative analysis within the Seurat package to eliminate batch effects during data integration. The data were successfully integrated (**Supplementary Figure S1D**), and then we employed uniform manifold approximation and projection (UMAP) to visualize the cell type distribution across each group (**Figure 1B**). Using reliable cell type-specific gene markers, we identified and annotated nine major cell types: fibroblasts (FB, marked by *PDGFRA*), endothelial cells (EC, marked by *VWF*), smooth muscle cells (SMC, marked by *ACTA2*), pericytes (PC, marked by *STEAP4* and *PDGFRB*), and immune cells, including *CD68*+ monocytes/macrophages (Mono/Mac, marked by *CD14*), T cells/Natural killer cells (T/NK, marked by *CD3D*), mast cells (MC, marked by *TPSB2*), plasma cells (Plasma, marked by *CD79A*), and Schwann cells (SWC, marked by *MPZ*) (**Figures 1B, C**, **Supplementary Figure S1G**). The distribution of these cell types was comparable across all samples (**Supplementary Figure S1E**). Each cell type exhibited a distinct gene expression profile, and through functional enrichment analysis of the top 100 cell type-specific marker genes, we elucidated the unique biological functions associated with each type (**Supplementary Tables S1**, **S2**; **Figure 1D**). For instance, FB were linked to “extracellular matrix organization” and “collagen fibril organization”, while SMC were associated with “muscle structure development” and “muscle contraction”. FB, EC, and SMC were the predominant cell types in penile tissue, consistent with previous studies (26) (**Figure 1E**). While the major cell type composition was largely consistent among samples, inter sample heterogeneity was evident (**Supplementary Figure S1F**). Notably, we observed increased percentages of Mono/Mac and T/NK cells in OsED, indicating an enhanced immune response in ARED (**Figure 1F**).

### 3.2 Global transcriptional changes in ARED

**Figure 2:**
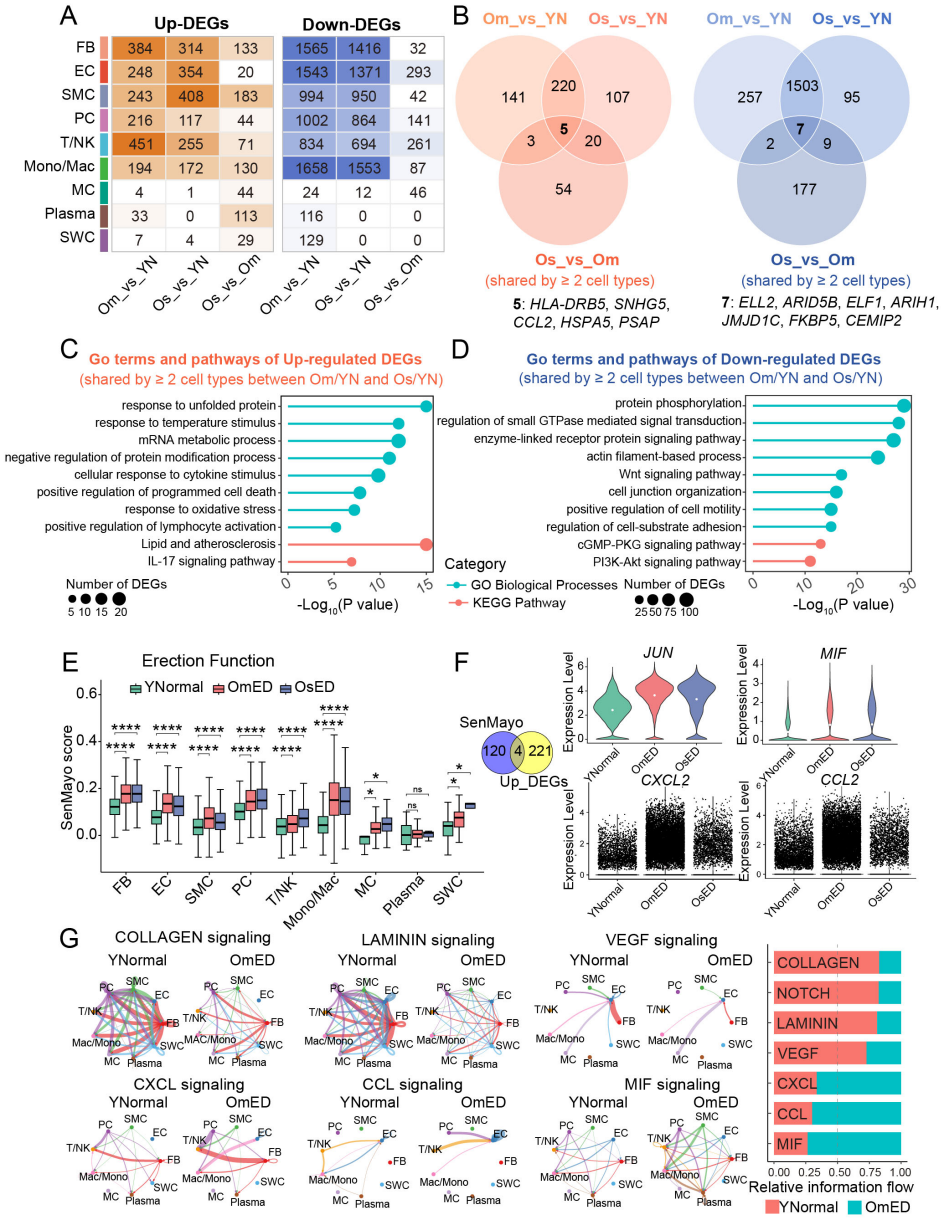
Changes in the transcriptional characteristics and cell-cell communications in ARED. **(A)** Heatmap showing the number of upregulated (left) and downregulated (right) DEGs per cell type derived from pairwise comparisons between groups. **(B)** Venn diagrams showing the overlaps of upregulated (left) and downregulated (right) DEGs (shared by ≥ 2 cell types) across three different group comparisons. Common upregulated (left) and downregulated (right) DEGs in pairwise comparisons are listed below. **(C, D)** Representative GO terms and pathways of common DEGs that are upregulated **(C)** and downregulated **(D)** in at least two cell types in both OmED and OsED compared with YNormal. **(E)** Box plot showing the SenMayo gene set score in different groups across different cell types ns, not significant; *, p.adj < 0.05; ****, p.adj < 0.0001. **(F)** Left, Venn diagram showing an overlap between SenMayo gene set and common upregulated DEGs. Right, Violin plots showing the expression levels of overlap genes in different groups. The white solid circle represents the mean value. **(G)** Circle plots showing the differential signaling pathways in intercellular communication network between YNormal and OmED (left). Stacked bar chart showing the overall information flow of these signaling pathways (right). Signal pathways colored red represent decreased in OmED and pathways colored green represent increased in OmED.

To investigate the transcriptomic changes associated with ARED, we analyzed differentially expressed genes (DEGs) (|avg_log2FC| > 0.25 and p_val_adj < 0.05) across various cell types that were aging-related or correlated with the severity of ED through pairwise differential expression analysis (Om_vs_YN: OmED vs. YNormal, Os_vs_YN: OsED vs. YNormal, Os_vs_Om: OsED vs. OmED). We found that FB, SMC, and T/NK cells exhibited the highest numbers of upregulated DEGs, while FB, EC, and Mono/Mac showed the largest number of downregulated DEGs. The number of DEGs in MC, Plasma, and SWC were smaller, likely due to their lower abundance (**Figure 2A**). Notably, almost all cell types exhibited significantly more downregulated DEGs than upregulated DEGs, indicating a decline in cellular function and a repression signature associated with ARED. The impairment or loss of cellular identity is a hallmark of cellular aging (27). Consistent with this, our data revealed a significant decline in cell identity scores for most cell types in the OmED group, with the most pronounced decline observed in EC (**Supplementary Figure S2A**). These findings suggest that EC are particularly vulnerable during penis aging.

Upset plots indicated that both the most upregulated and downregulated DEGs were specific to particular cell types (**Supplementary Figures S2B, C**). We classified DEGs that were affected in two or more cell types as genes whose expression changed across all cell types in ARED. Specifically, we identified 369 DEGs in the Om_vs_YN group and 352 DEGs in the Os_vs_YN group that were consistently upregulated in at least two cell types. Additionally, there were 1,769 downregulated DEGs in the Om_vs_YN group and 1,614 in the Os_vs_YN group (**Figure 2B**). Notably, approximately two-thirds of the upregulated DEGs in the Os_vs_YN group were also detectable in the Om_vs_YN group, while about 90% of the downregulated DEGs in the Os_vs_YN group were present in the Om_vs_YN group. This suggests that substantial transcriptional changes had already occurred in individuals with mild erectile dysfunction. As the condition progresses, five DEGs (*HLA-DRB5*, *SNHG5*, *CCL2*, *HSPA5*, and *PSAP*) showed sustained upregulation, while seven DEGs (*ELL2*, *ARID5B*, *ELF1*, *ARIH1*, *JMJD1C*, *FKBP5*, and *CEMIP2*) exhibited consistent downregulation (**Figure 2B**).

Next, we conducted functional enrichment analysis to investigate the core pathways enriched among the DEGs shared across different cell types (**Supplementary Table S3**). The 225 DEGs that were co-upregulated in at least two cell types in the OmED and OsED groups were primarily associated with immune and inflammatory responses, including terms like “cellular response to cytokine stimulus”, “positive regulation of lymphocyte activation”, and “IL-17 signaling pathway” (**Figure 2C**). Additionally, gene ontology (GO) terms such as “positive regulation of programmed cell death” and “response to oxidative stress” were also enriched among these upregulated DEGs. In contrast, the 1,510 common downregulated DEGs were largely related to cell structure and function, with pathways including “actin filament-based process,” “cGMP-PKG signaling pathway” and “cell junction organization” (**Figure 2D**). Furthermore, pathways associated with cell proliferation and survival, such as the “PI3K-Akt signaling pathway” were also enriched.

SenMayo, a previously validated gene set, increases with age across various tissues and can be used to identify cells that express high levels of senescence/SASP genes (25). We utilized the SenMayo list to compute the senescence/SASP score for each cell type across different erectile function groups. We found that nearly all cell types in the ARED group exhibited significantly higher gene set scores compared to the YNormal group (**Figure 2E**). By combining the analysis of SenMayo genes with the 225 common upregulated DEGs, we identified key genes—including *JUN*, *MIF*, *CXCL2*, and *CCL2*—that may contribute to cellular senescence and heightened inflammatory responses in ARED (**Figure 2F**).

To investigate dynamic cell communication in both normal and ARED, we conducted cell-cell interaction analysis using CellChat. We identified 2,238 interactions in the YNormal group, 1,052 in the OmED group, and 794 in the OsED group, with interaction strength decreasing in the OmED and OsED groups (**Supplementary Figure S2D**). Strong interactions were primarily observed among stromal cells, particularly between FB and EC; however, these interactions diminished with aging, while the number of differential interactions involving certain immune cells increased in ARED (**Supplementary Figure S2F**). Comparing the outgoing and incoming interaction strength revealed that FB exhibited the strongest outgoing interaction strength, aligning with previous findings (26) and underscoring their crucial role in the microenvironment of penile tissue (**Supplementary Figure S2E**). Additionally, we analyzed the changes in signaling pathways by comparing the overall information flow of each pathway between the YNormal and OmED groups. Signaling related to COLLAGEN and LAMININ, part of ECM-receptor interactions, was reduced with aging. Furthermore, NOTCH and VEGF signaling, which are associated with vascular tissue regulation and angiogenesis (28), also declined (**Figure 2G**). In contrast, CCL, CXCL, and MIF signaling pathways, linked to inflammatory responses, were further activated in the OmED group.

### 3.3 SMC displayed relaxation and contraction dysfunction in ARED

**Figure 3:**
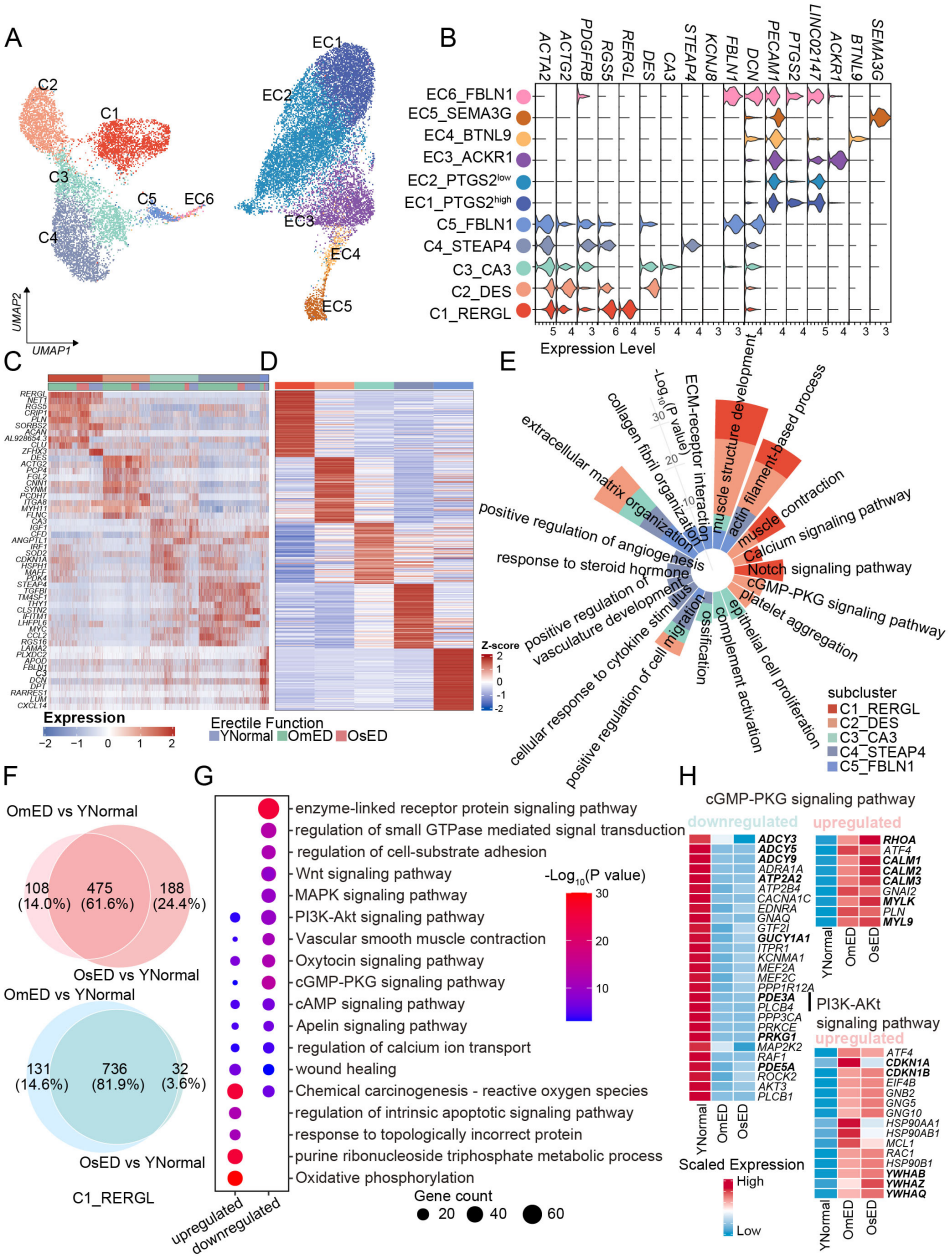
Transcriptional and functional heterogeneity within SMC subclusters and between different groups. **(A)** UMAP plot showing the different SMC, PC and EC subclusters in human penile CC. **(B)** Violin plot showing the marker genes of each subclusters. **(C)** Top 10 DEGs of SMC and PC subclusters, for subcluster color code see **(A)**. **(D)** Heatmap showing top 100 marker genes of each subclusters based on YNormal group. **(E)** Circular bar plot showing the pathways associated with marker genes in **(D)**. **(F)** The Venn diagrams showing the number of upregulated genes (top) and downregulated DEGs (bottom) shared between OmED vs YNormal and OsED vs YNormal in C1_RERGL. **(G)** Representative pathways of overlapped upregulated (475) and downregulated (736) DEGs in C1_RERGL. **(H)** Heatmap showing the relative expression levels of genes related to cGMP-PKG signaling pathway in C1_RERGL across different groups. **(I)** Heatmap showing the relative expression levels of upregulated genes related to PI3K-Akt signaling pathway in C1_RERGL across different groups.

Physiological penile erection relies on the interaction between EC and SMC. Pericytes, also known as perivascular cells, play a role in the pathogenesis of erectile function (29) and, as our results suggest (**Figure 1D**), are involved in angiogenesis and muscle system processes. In this study, we analyzed these three cell types together. Following re-clustering and UMAP analysis, SMC and pericytes were classified into five subclusters (C1-C5), while EC were divided into six subclusters (EC1-EC6) (**Figure 3A**). These subclusters included cells from both YNormal and ARED patients, but the UMAP plot revealed significant heterogeneity between the penile tissues of the two groups (**Supplementary Figure S3A**). In addition to expressing genes associated with SMC or EC identity, such as *ACTA2* and *PECAM1*, each subcluster exhibited its own distinct transcriptomic profile (**Figures 3B, C**).

In our further analysis of the SMC subclusters, we identified 320, 341, 227, 408, and 218 DEGs in the YNormal group for each respective subcluster. Column clustering indicated that C1_RERGL was closer to C2_DES, suggesting that these two subgroups may share similar transcriptomic features and functions compared to the other subclusters (**Supplementary Figure S3B**). Differential expression and functional enrichment analyses of the top 100 DEGs from each subcluster revealed that C1_RERGL and C2_DES were associated with “muscle structure development” and “muscle contraction” exhibiting high expression of contractile-related genes such as *MYH11* and *MYL9* (**Figures 3D, E**; **Supplementary Figure S3C**). Additionally, the “Notch signaling pathway” was enriched in C1_RERGL, while “positive regulation of vasculature development” was enriched in C4_STEAP4, indicating their roles in vascular development (**Figure 3E**). C5_FBLN1 exhibited the highest ECM score (**Supplementary Figure S3D**), which included core extracellular matrix components like collagen, glycoprotein, and proteoglycan (30). The DEGs in C5_FBLN1 included matrix and collagen-related genes, such as *FBLN1* and *LUM*, and were enriched in pathways like “collagen fibril organization” and “ECM-receptor interaction”. These findings indicate that C5_FBLN1 exhibits functional characteristics of fibroblasts, defining it as a fibroblast-like subcluster (**Figures 3B, E**). Based on the distribution of subcluster markers (*DES*, *THY1*, and *LUM*) (**Supplementary Figure S3E**), which have been validated through IHC and immunofluorescence co-staining in human corpora cavernosa tissue (26), we infer that C1_RERGL consists of vascular SMC, C2_DES and C3_CA3 are trabecular SMC of the cavernosum, and C4_STEAP4 represents pericytes.

To further characterize the changes in SMC associated with ARED, we examined the transcriptional alterations within each SMC subcluster. Our analysis revealed that the DEGs and enriched pathways in the OmED group largely overlapped with those in the OsED group when compared to the YNormal group across various subclusters (**Figure 3F**; **Supplementary Figure S4A**). Here, we focused on the common transcriptomic changes shared between the OmED and OsED groups relative to the YNormal group to identify commonalities in aging-related ED. Differential expression analysis identified 475 upregulated genes and 736 downregulated genes in the C1_RERGL subcluster (**Figure 3F**). The top upregulated GO terms related to ARED included “oxidative phosphorylation” and “purine ribonucleoside triphosphate metabolic process” both associated with energy metabolism (**Figure 3G**). In contrast, the downregulated DEGs were primarily enriched in GO terms such as “enzyme-linked receptor protein signaling pathway” and “regulation of small GTPase-mediated signal transduction”.

A normal penile erection requires the relaxation of smooth muscle and the accumulation of blood in the CC by increasing arterial inflow while blocking venous outflow (31). At the molecular level, nitric oxide (NO) serves as the primary mediator of penile smooth muscle relaxation, activating the guanylate cyclase (GC)-cGMP pathway to lower intracellular calcium levels, which interferes with the contraction of cavernous smooth muscle and promotes relaxation (32). Our analysis revealed a significant decrease in the cGMP-PKG signaling pathway in ARED (**Figure 3G**). We observed reduced expression of *GUCY1A1*, which encodes an alpha subunit of soluble guanylate cyclase (sGC) responsible for converting GTP to the second messenger cGMP (**Figure 3H**). Consequently, cGMP levels may also be lower in OmED, and the expression of the downstream protein kinase *PRKG1*, a key mediator in the NO/cGMP signaling pathway, was also diminished (**Figure 3H**; **Supplementary Figure S3F**). Interestingly, we noted a decrease in the expression of PDE5A in OmED, potentially explaining the poor response to oral PDE5 inhibitors in some ED patients (**Figure 3H**; **Supplementary Figure S3F**). Another pathway to reduce intracellular calcium levels involves cyclic adenosine monophosphate (cAMP). In the OmED group, we found decreased expression of adenylate cyclase (*ADCY3*, *ADCY5*, and *ADCY9*), which catalyzes the formation of cAMP, as well as decreased expression of *PDE3A*, which terminates the effects of cAMP (**Figure 3H**). Additionally, *PRKACB*, which encodes a catalytic subunit of cAMP-dependent protein kinase, and *ATP2A2*/*SERCA2*, an ATPase responsible for transporting intracellular calcium ions into the sarcoplasmic reticulum to lower intracellular free calcium levels (33), were also found to be reduced (**Figure 3H**, **Supplementary Figure S3F**).

In contrast to the impaired smooth muscle relaxation seen in ARED, the contraction of smooth muscle may actually be enhanced (**Supplementary Figure S3G**). Specifically, alongside the upregulation of calmodulin-encoding genes (*CALM1*, *CALM2*, and *CALM3*), expression of smooth muscle contraction-related myosin light chain genes (*MYL6*, *MYL9*) (34) was also elevated in the ARED group. Concurrently, an increase in the expression of *RHOA* within the RHO/ROCK signaling pathway was observed (**Figure 3H**; **Supplementary Figure S3H**). Additionally, the expression of alpha adrenoceptors involved in contraction pathways displayed variability among different subclusters and groups. *ADRA2A* was primarily expressed in C1_RERGL, while *ADRA2C* was mainly found in C2_DES, with both showing increased expression levels in the OmED group (**Supplementary Figure S3I**).

In addition to the DEGs and signaling pathways related to smooth muscle relaxation and contraction, we also noticed other different signaling pathways between ARED and YNormal. “Wnt signaling pathway”, “MAPK signaling pathway” and “PI3K-Akt signaling pathway” were declined/inhibited in ARED (**Figure 3G**). It is well known that PI3K/Akt pathway plays an important role in promoting cell survival, cell cycle progression and cell proliferation in various cell types, such as vascular SMCs (35). *CDKN1A* (p21) and *CDKN1B* (p27), potent cyclin-dependent kinase inhibitor, were upregulated in ARED (**Figure 3I**). FOXO3 is downstream target of the PI3K/AKT pathway (36) and a convincing longevity gene, its expression decreased in ARED, which might be inhibited by the members of 14-3-3 proteins family (*YWHAB*, *YWHAZ* and *YWHAQ*) (**Figure 3I**, **Supplementary Figure S3J**).

### 3.4 Endothelial functions declined in ARED

To investigate the functional changes in EC, we began with a differential expression gene analysis. Our findings revealed that in the ARED group, 228 genes were consistently upregulated, while 1,257 genes were consistently downregulated (**Supplementary Figures S5A, B**). Subsequent pathway enrichment analysis showed that the upregulated genes were primarily associated with immune-related signaling pathways, including “Antigen processing and presentation”, “Interferon alpha/beta signaling”, “cellular response to cytokine stimulus” and “positive regulation of leukocyte activation”. Additionally, pathways related to “Platelet degranulation” and “Platelet activation, signaling, and aggregation” were also enriched (**Supplementary Figure S5C**). In contrast, the downregulated genes were linked to pathways such as “cell junction organization” and “endothelium development” (**Supplementary Figure S5D**). These results suggest endothelial dysfunction.

Compared to the YNormal group, nearly half of the upregulated DEGs in the OmED and OsED groups did not overlap, suggesting their involvement in distinct biological processes. We performed pairwise comparisons (OmED vs. YNormal, OsED vs. YNormal, OsED vs. OmED) and merged the upregulated DEGs from these comparisons into a single candidate gene set. Clustering analysis revealed two specific gene clusters—C2 and C4—that exhibited progressively increased expression with worsening ED severity (**Supplementary Figure S5E**). Notably, the C4 cluster showed a significant increase in expression in the OmED group, while the C2 cluster exhibited more pronounced changes in the OsED group. Enrichment analysis indicated that the C4 cluster was primarily associated with biological processes such as “regulation of mRNA metabolic process”, “oxidative phosphorylation” and “DNA damage response”. In contrast, the C2 cluster was more linked to immune-related processes, suggesting a heightened immune response in the OsED group (**Supplementary Figure S5F**).

### 3.5 Transcriptomic and metabolic heterogeneity of EC in normal and ARED

**Figure 4:**
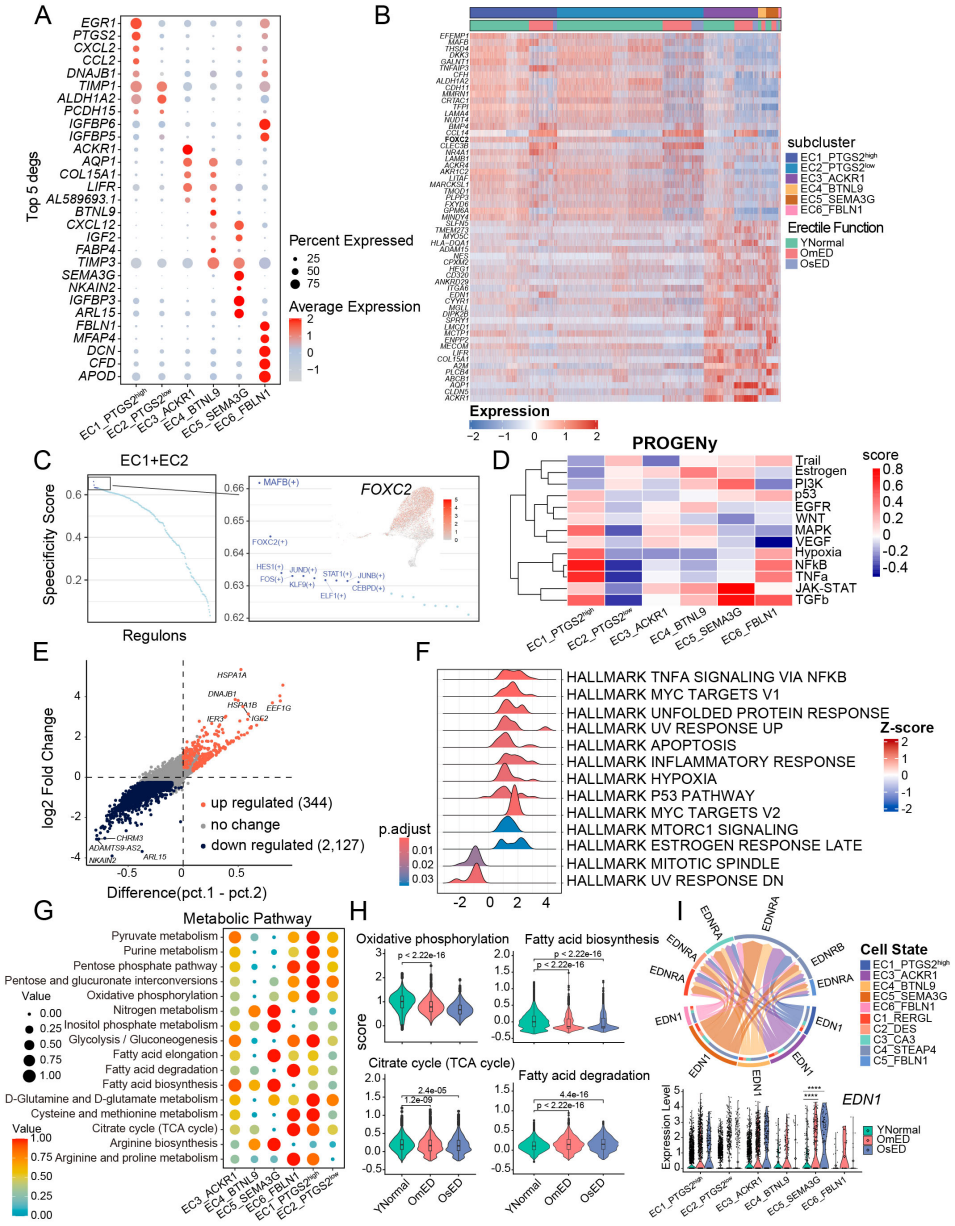
Heterogeneity in EC subclusters and ARED. **(A)** Bubble diagram showing the expression of top 5 marker genes for each EC subclusters. **(B)** Heatmap showing the expression profile of differentially expressed genes in CC EC (EC1+EC2) versus pan-vascular EC (EC3+EC4+EC5). **(C)** Dot plot showing the TF regulons in CC EC. The top 10 TFs are highlighted (right). UMAP showing the distribution and the expression of *FOXC2* (right). Red color indicates high expression levels. **(D)** Heatmap showing the mean pathway activity scores of different EC subclusters. **(E)** Scatter plot showing the expression changes of all genes in OmED compared to YNormal in EC5_SEMA3G. Genes significantly upregulated in OmED are highlighted in tomato color, whereas genes downregulated are marked in dark color. No changed genes are marked in grey. **(F)** Ridge plots showing the results of Gene set enrichment analysis (GSEA). Compared with YNormal, the significant HALLMARK pathways enriched in OmED EC5_SEMA3G. DEGs in **(E)** used. **(G)** Dot plot of main metabolic characteristics in each EC subclusters, both size and color indicate the effect size. **(H)** Violin and box plots showing the activity scores of Oxidative phosphorylation, Citrate cycle (TCA cycle), Fatty acid biosynthesis and Fatty acid degradation in EC across different groups. Box shows the median and the quartile range (25%-75%). P values by Wilcoxon test are indicated. **(I)** Specific signaling ligand-receptors pairs showing in chord diagram (top). Violin plot of the expression levels of *EDN1* in the six EC subclusters and different groups (bottom). **** p.adj < 0.0001 (two-sided Wilcoxon rank-sum test).

To investigate the heterogeneity of EC and identify any unique EC subclusters within these groups, we conducted sub-clustering analysis. This resulted in the identification of six EC subclusters: EC1_PTGS2^high^, EC2_PTGS2^low^, EC3_ACKR1, EC4_BTNL9, EC5_SEMA3G, and EC6_FBLN1 (**Figure 3A**; **Supplementary Figures S6A, B**). We then identified marker genes for each subcluster and annotated them accordingly (**Figure 4A**).

EC1_PTGS2^high^ exhibited high expression of *EGR1*, a gene that can be rapidly induced by various stimuli and is involved in regulating the expression of several vascular genes (37, 38). Additionally, this subcluster expressed *PTGS2*, which plays a role in inflammatory responses and angiogenesis in various disorders (39), along with chemokines *CXCL2* and *CCL2*, indicating its involvement in the progression of vascular diseases (**Figure 4A**). In EC3_ACKR1, *ACKR1* was predominantly expressed, which is known to be highly present in small venules rather than in arteries, capillaries, or most lymphatics (40). EC4_BTNL9 expressed the capillary-related gene *BTNL9*. Exclusively, *SEMA3G* was found in EC5_SEMA3G, where it is expressed in major and small branching arteries (41). EC6_FBLN1 showed high levels of *FBLN1* and *DCN*, both of which are extracellular matrix (ECM) proteins involved in ECM organization and regulation of pro-inflammatory processes and fibrillogenesis, suggesting a potential endothelial-mesenchymal transition (42). EC3_ACKR1, EC4_BTNL9, and EC5_SEMA3G exhibited relatively high pan-vascular scores, categorizing them as vascular EC (**Supplementary Figure S6C**). Notably, in addition to their subcluster-specific markers, these three EC subclusters also expressed classic vascular endothelial subtype markers (**Supplementary Figure S6D, F**), which allowed for further annotation. EC3_ACKR1 included genes related to leukocyte adhesion, such as *ICAM1*, *SELE*, and *SELP* (40), suggesting they may represent venous EC. The capillary marker *RGCC* was primarily expressed in EC4_BTNL9 (43). EC5_SEMA3G displayed a strong transcriptional signature of arterial ECs, with expression of *GJA4* and *GJA5*, which encode tight and gap junction proteins, as well as *FBLN5* and *FBLN2*, which encode ECM proteins that contribute to blood vessel wall elastogenesis and arterial stiffness (44). Additionally, transcription factors *HEY1* and *SOX17* were also expressed in this subcluster (**Supplementary Figure S6D, F**), confirming that EC5_SEMA3G consists of arterial EC.

EC1_PTGS2^high^ and EC2_PTGS2^low^ both expressed *KIT* (**Supplementary Figure S6E**), which is primarily found in cavernosal trabecular EC rather than vascular EC (26), highlighting their unique transcriptional characteristics. By comparing differential genes with vascular EC, we identified 29 genes that were significantly upregulated in both EC1_PTGS2^high^ and EC2_PTGS2^low^ (**Figure 4B**). Subsequent SCENIC analysis indicated the presence of several key transcription factors in the EC1 and EC2 subclusters (**Figure 4C**). Among them, *MAFB* and *FOXC2*, are known to play roles in lymphangiogenesis, endothelial specification, and vascular repair (45).

We conducted pathway analysis on each EC subcluster using PROGENy (46). The JAK-STAT and PI3K signaling pathways were upregulated in EC5_SEMA3G, while EC1_PTGS2^high^ and EC6_FBLN1 showed increased activity in Hypoxia, NFkB, and TNFa pathways. This further corroborated the involvement of EC1 and EC5 in immune response and endothelial dysfunction (**Figure 4D**). As blood flows into the corporal sinusoids through the arterial vessels, we noted the heterogeneity of EC5_SEMA3G in ARED. The differential analysis between the OmED and YNormal groups revealed 344 upregulated DEGs and 2,127 downregulated DEGs (**Figure 4E**). Gene Set Enrichment Analysis (GSEA) of these DEGs identified enrichment in pathways such as “HALLMARK TNFA SIGNALING VIA NFKB”, “HALLMARK APOPTOSIS”, “HALLMARK INFLAMMATORY RESPONSE”, and “HALLMARK HYPOXIA” in the OmED group (**Figure 4F**). Additionally, the APOPTOSIS score increased across all subclusters in the OmED group, with the expression of *TXNIP*, which induces apoptosis under oxidative stress (47), elevated in ARED patients (**Supplementary Figure S6G**). The expression of *ATF4*, *HSPB1*, *MCL1*, *PPIA*, *SOD1*, and *RACK1*—genes associated with the “regulation of oxidative stress-induced intrinsic apoptotic signaling pathway”—also rose in OmED EC (**Supplementary Figure S6H**). Furthermore, Gene Set Variation Analysis (GSVA) across all genes in the six subclusters indicated increased activity in various signaling pathways related to “Chemical carcinogenesis – reactive oxygen species”, “Complement and coagulation cascades” and “Lipid and atherosclerosis” (**Supplementary Figure S6I**). Collectively, these findings suggest that enhanced immune response and apoptosis are key features of ARED EC, manifesting even in cases of mild ED.

EC metabolism is closely linked to their functions, such as angiogenesis. To further investigate the metabolic profiles of EC subclusters in the penis, we used the R package “scMetabolism” (48) to quantify metabolic activity (**Figure 4G**). The “Glycolysis/Gluconeogenesis” pathway was enriched in EC1_PTGS2^high^, EC3_ACKR1, and EC6_FBLN1, while the “Oxidative Phosphorylation” (OXPHOS) pathway was particularly active in EC1_PTGS2^high^. EC5_SEMA3G displayed higher activity in “Nitrogen metabolism”, “Inositol phosphate metabolism”, “Fatty acid elongation” and “Fatty acid biosynthesis”. Additionally, “Arginine biosynthesis” was prominent in vascular EC (EC3_ACKR1, EC4_BTNL9, and EC5_SEMA3G), facilitating the production of L-arginine and the synthesis of the endothelium-derived relaxing factor NO (49). We also examined changes in metabolic pathway activity between the YNormal and ARED groups. OXPHOS and the “Citrate Cycle (TCA cycle)” are key pathways for ATP production linked to mitochondrial function (50). Our analysis revealed reduced activity in both OXPHOS and the TCA cycle in ARED (**Figure 4H**), indicating mitochondrial dysfunction. This OXPHOS impairment, which induces oxidative stress, may contribute to endothelial injury in the penis affected by ARED. Notably, while fatty acids are a major energy source for many tissues, we observed that ARED EC exhibited increased “Fatty acid degradation” and significantly decreased “Fatty acid biosynthesis” compared to the YNormal group (**Figure 4H**). Overall, our findings highlight metabolic heterogeneity both within EC subclusters and between ED groups.

Endothelin-1 (ET-1/EDN1) is a potent vasoconstrictor peptide and one of the first endothelium-derived contracting factors (51). Analysis of the cellular ligand-receptor network for the EDN pathway indicated that EC5_SEMA3G acted as the strongest sender, while C4_STEAP4 demonstrated higher signal reception (**Figure 4I**), mediating the contractile response of pericytes. Notably, the expression of *EDN1* was elevated in ARED, particularly in EC5_SEMA3G.

### 3.6 Penile immune microenvironment exhibited pro-inflammatory traits

**Figure 5:**
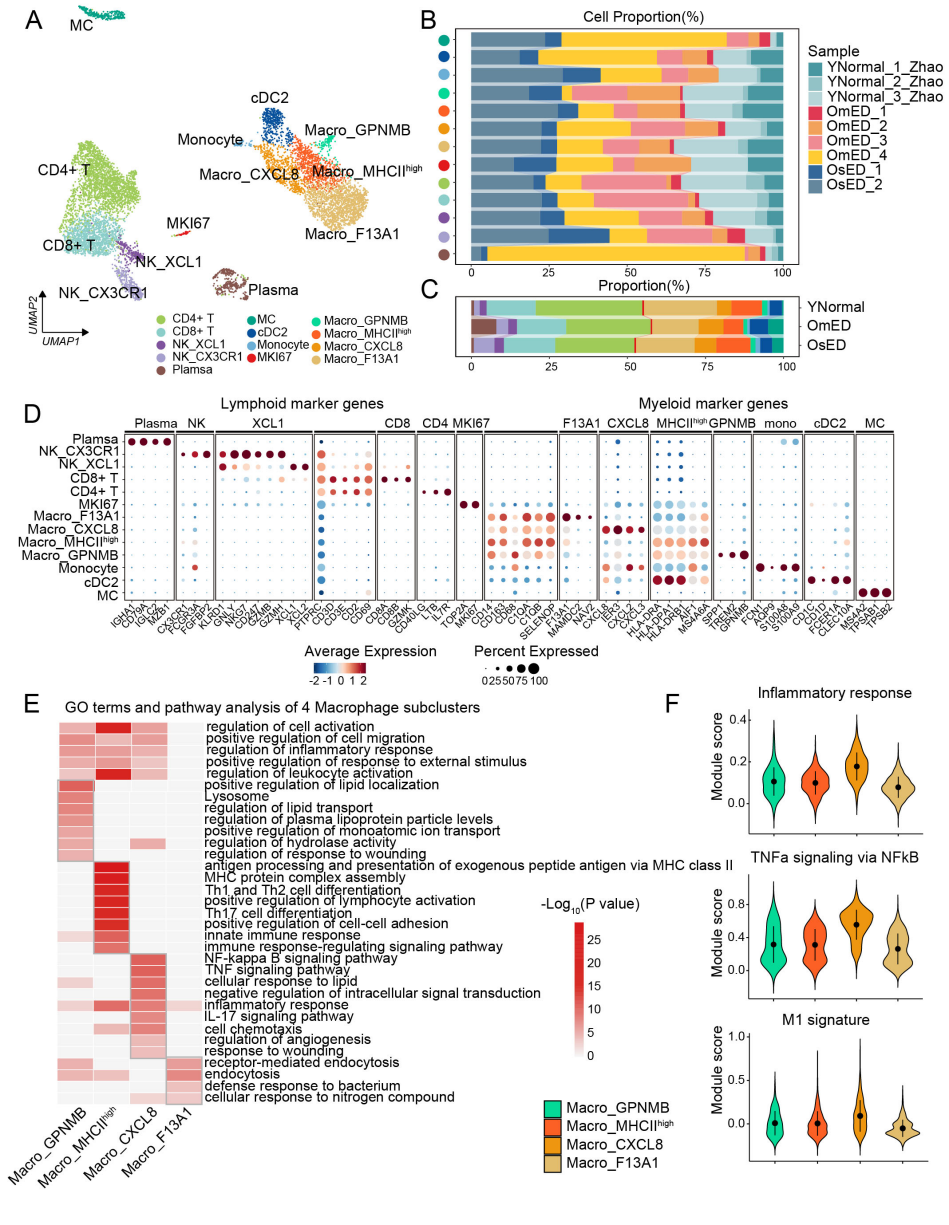
Composition of the immune microenvironment in human penile CC. **(A)** UMAP plot showing 13 immune subclusters. **(B-C)** Bar plot showing the proportion of each immune subclusters in each sample **(B)** and each group **(C)**. **(D)** Bubble diagram showing marker genes across 13 immune subclusters. **(E)** Heatmap showing GO terms and pathways of marker genes in four Macro subclusters. **(F)** Gene set score analysis of “Inflammatory response”, “TNFa signaling via NFkB”, and “M1 signature” in four Macro subclusters.

Immune cells, alongside stromal cells, represent a significant population in penile tissue. UMAP and clustering analysis revealed that this tissue primarily consists of myeloid and lymphoid immune cells (**Figure 1B**). To investigate the role of immune cells in ARED, we subset and re-clustered all immune cells from both normal and ARED samples, identifying various clusters including macrophages, monocytes, cDC2, mast cells, T cells, NK cells, Plasma cells, and a small group of proliferating immune cells based on characteristic marker genes and gene signatures (**Figures 5A, D**; **Supplementary Figures S7A, B**).

While some immune cell subclusters displayed heterogeneity based on their sample origin, they included both YNormal and ARED-derived cells and transcriptomes, highlighting changes in the cellular composition of the penile microenvironment (**Figures 5B, C**, **Supplementary Figure S7C**). Within the lymphoid compartment, the CD4+ T cells, likely suppressive Treg cells characterized by the expression of immune-inhibitory [*BTLA*, *CTLA4*, *PDCD1* (52)], and Treg markers (*IL2RA, FOXP3*), were found in a decreased proportion in ARED (**Figure 5C**; **Supplementary Figure S7D**). *CD8*+ T cells also expressed *PDCD1* and *LAG3*, indicating an exhaustion signature (53) (**Supplementary Figure S7D**). We identified two NK subclusters with distinct transcriptional characteristics and functions: NK_XCL1, which expressed chemokines *XCL1* and *XCL2*, and NK_CX3CR1, which expressed *CX3CR1*, *FCGR3A*, *FGFBP2*, and genes encoding cytotoxic molecules such as *GZMH*, *GZMA*, *GZMB*, *NKG7*, *GNLY*, and *PRF1* (**Figure 5D**; **Supplementary Figures S7D-F**). Although the proportion of NK_CX3CR1 increased in ARED (**Figure 5C**; **Supplementary Figure S7C**), its cytotoxicity score was reduced in the OmED group (**Supplementary Figure S7G**).

**Figure 6:**
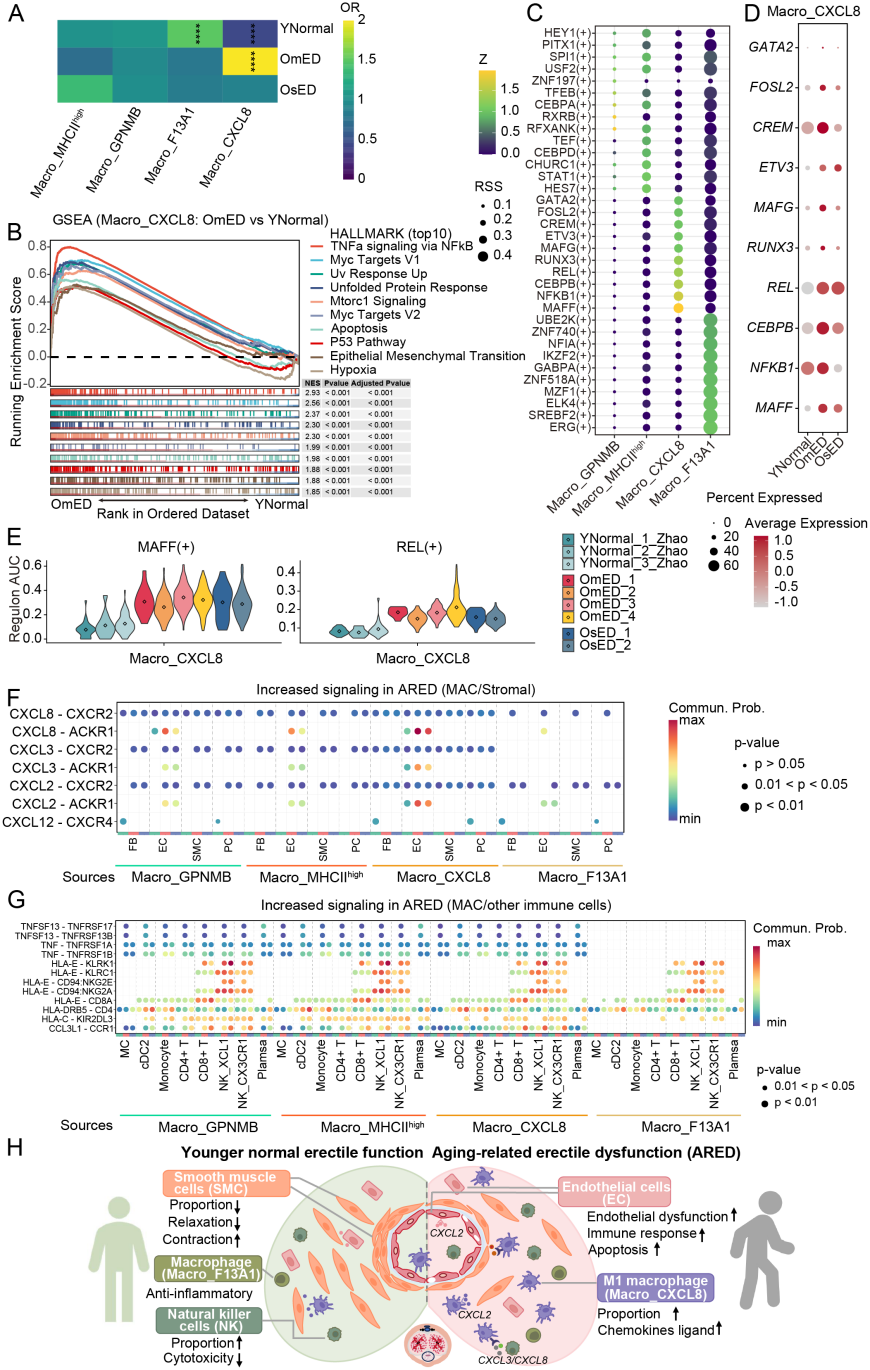
Alterations of Macro_CXCL8 in ARED and cell-cell communications.**(A)** The heatmap displaying the ORs of four macrophage subclusters occurring in each group. OR > 1.5 indicates that the subcluster is more likely to be distributed in the corresponding group. **** represents adjust P_value < 0.0001 and OR > 1.5 or OR < 0.5. **(B)** Gene set enrichment analysis (GSEA) showing HALLMARK pathways upregulated in OmED Macro_CXCL8 compared to YNormal Macro_CXCL8. **(C)** Bubble diagram showing the top 10 TF regulons of four Macro subclusters ordered by regulon-specific score (RSS). **(D)** Bubble diagram showing the expression levels of Macro_CXCL8 top 10 specific TF in different groups. **(E)** Regulon AUC scores of Macro_CXCL8 specific regulons across each sample. **(F)** Bubble plot showing the up-regulated signaling ligand-receptor pairs in ARED between four Macro subclusters and stromal cells. **(G)** Bubble plot showing the up-regulated signaling ligand-receptor pairs in ARED between four Macro subclusters and other immune cells. **(H)** A schematic illustration showing the dynamics of cellular dysfunction and molecular signaling in ARED. .

Among myeloid cells, we identified MC and cDC2, a subset of conventional dendritic cells (cDCs), which expressed markers such as *CD1C*, *CD1D*, *FCER1A*, and *CLEC10A* (54). We also found a small group of monocytes characterized by *FCN1*, *S100A8*, and *S100A9* (**Figure 5D**). Additionally, we categorized four subclusters of macrophages (Macro), the predominant innate immune cells: Macro_GPNMB, Macro_MHCII^high^, Macro_CXCL8, and Macro_F13A1. We computed marker genes, and the expression of DEGs in each macrophage subcluster is illustrated in the heatmap (**Supplementary Figure S7H**). Pathway enrichment analysis revealed that the marker genes in several macrophage subclusters were associated with similar functions, including “regulation of cell activation”, “regulation of inflammatory response”, “positive regulation of response to external stimulus” and “regulation of leukocyte activation” highlighting their roles in immune responses (**Figure 5E**; **Supplementary Table S4**). Furthermore, the DEGs in each macrophage subcluster were enriched in specific GO terms and pathways, indicating functional diversity. For instance, “positive regulation of lipid localization” and “regulation of lipid transport” were enriched in Macro_GPNMB; “antigen processing and presentation of exogenous peptide antigen via MHC class II” and “MHC protein complex assembly” were specific to Macro_MHCII^high^; Macro_CXCL8 was enriched in the “NF-Kappa B signaling pathway”, “TNF signaling pathway”; while Macro_F13A1 showed enrichment in “defense response to bacterium”. Macro_CXCL8 exhibited high expression of pro-inflammatory genes such as *IL1A* and *IL1B*, along with elevated scores for “Inflammatory response”, “TNFa signaling via NFkB” and “M1 signature” (**Figure 5F**; **Supplementary Figure S7I**). In contrast, Macro_F13A1 expressed higher levels of anti-inflammatory genes, displayed low scores for immune response signatures, and had elevated scores for “Phagocytosis” (**Figure 5F**; **Supplementary Figures S7I-J**). These findings suggest that macrophages had both pro-inflammatory and anti-inflammatory states within the penile microenvironment. The proportion of pro-inflammatory Macro_CXCL8 was found to be increased in ARED (**Figure 5C**). And using the odds ratio (OR) analysis (55), we found anti-inflammatory Macro_F13A1 was enriched in YNormal, Macro_CXCL8 appeared to be OmED-enriched (**Figure 6A**), indicating an inflammatory activation. Collectively, these observations suggest that the enrichment of NK_CX3CR1 and Macro_CXCL8 may contribute to the pathogenesis of ARED.

### 3.7 Macro_CXCL8 activated during aging

We then concentrated on the Macro_CXCL8 subcluster, known for its M1 pro-inflammatory characteristics, to investigate changes during the progression of ARED. GSEA indicated the activation of inflammatory pathways in the OmED group, including “TNFa signaling via NFkB”, “Inflammatory Response” and “Interferon Gamma Response” along with pathways related to “Hypoxia” and “Glycolysis” (**Figure 6B**; **Supplementary Figure S8B**). Hypoxia alters the inflammatory microenvironment and promotes M1 macrophage polarization (56). Additionally, enhanced glycolytic metabolism is a hallmark of M1 macrophage activation, supporting cell viability and inflammatory activity (57). The pathways related to “Interferon Gamma Response” and “Interferon Alpha Response” were further upregulated in the OsED group (**Supplementary Figure S8C**). We observed increased expression of pro-inflammatory factors such as *IL1B*, *CCL4*, and *CXCL3* compared to the YNormal group (**Supplementary Figure S8A**). Moreover, the upregulated DEGs in ARED were enriched in numerous disease-related KEGG pathways, including “Lipid and Atherosclerosis” and “Rheumatoid Arthritis” underscoring the significant role of these pro-inflammatory macrophages in the onset and progression of ARED, likely by contributing to inflammatory processes and vascular alterations (**Supplementary Figure S8D**).

We then conducted SCENIC analysis to investigate the mechanisms driving M1 macrophage activation in ARED. Each Macro subcluster exhibited specific TFs (**Figure 6C**). Notably, the TF activity and gene expression levels in Macro_CXCL8 were both elevated in ARED, with particularly pronounced changes observed for MAFF and REL (**Figures 6D, E**). The NF-kB pathway plays a crucial role in M1 macrophage polarization via TLR signaling (58). And the p50 and c-Rel proteins, encoded by *NFKB1* and *REL*, are essential components of the NF-kB family that can form heterodimers in response to various stimuli, initiating the transcription of multiple genes (59). TF MAFF is linked to inflammation and lipid metabolism (60). The upregulated target genes influenced by REL(+) and MAFF(+) included chemokines such as *CXCL2* and *CXCL8*, along with Zinc Finger Protein 91 (*ZFP91*), which promotes the production of the inflammatory cytokine IL-1β in macrophages by activating the MAPK pathway and atypical caspase-8 inflammasome (61) (**Supplementary Figure S8E**). These upregulated genes were enriched in immune-related pathways (**Supplementary Figure S8F**). Overall, our findings suggest that these TFs may significantly impact M1 macrophage activation.

### 3.8 Cell communication between four Macro and other cell types

To better understand the biological characteristics of macrophage interactions with other cell types, we performed CellChat analysis to infer intercellular communication. The results indicated that most signals emitted by macrophages were primarily received by other immune cells and EC (**Supplementary Figure S9A**). Notably, several immune-related signaling pathways, including “MHC-I”, “MHC-II”, “CCL” and “TNF”, were enriched across the four macrophage subclusters. Additionally, signaling through “SPP1” and “APOE” was uniquely enriched in the Macro_GPNMB subcluster (**Supplementary Figure S9B**). By comparing overall communication probabilities from macrophages to stromal cells and other immune cells between the different groups, we identified several active pathways in ARED. We observed that chemokines *CXCL2*, *CXCL3*, and *CXCL8* could interact with EC via CXCL2/CXCLs-ACKR1 pathways (**Figure 6F**). In ARED, the expression levels of *CXCL2*, *CXCL3*, and *CXCL8* in macrophages were elevated, and the receptor ACKR1 in EC, particularly in EC3_ACKR1, also showed increased expression (**Supplementary Figure S9D**). Furthermore, we noted interactions between macrophages and CD8+ T cells, as well as NK cells, through HLA-E–CD94:NKG2A, HLA-E–KLRK1, HLA-E–CD94:NKG2E, and HLA-E–KLRC1 ligand-receptor pairs (**Figure 6G**). The immunosuppressive signal HLA-E–CD92:NKG2A has been linked to promoting the accumulation of senescent cells (62) and affecting NK cell cytotoxicity (63). HLA-E–KLRK1 interaction was observed enhanced in ARED (**Figure 6G**). EC also engaged closely with NK cells through these signals (**Supplementary Figure S9C**). In ARED, expression levels of both the ligand HLA-E and its receptors increased, with HLA-E distributed across all cell types and its receptors predominantly expressed in *CD8*+ T and NK cells (**Supplementary Figure S9E**). Additionally, EC communicated with other immune cells through ligand-receptor pairs such as CCL14–CCR1, and CCL2–CCR2 (**Supplementary Figure S9C**). Chemokines CCL2 are particularly involved in acute inflammation and the recruitment of monocytes and eosinophils (64), while CCL14 binds to CCR1, regulating inflammatory cell infiltration after injury (65). These findings deepen our understanding of the progression of ARED.

## 4 Discussion

The human penis is a complex tissue structure. Recent groundbreaking studies have utilized single-cell resolution to describe its cellular composition, uncovering potential molecular mechanisms associated with vasculogenic ED (26, 66, 67). Among these studies, Zhao et al. focused on relatively young ED patients (< 56 years old), systematically characterizing the heterogeneity and spatial distribution of FB, SMC and EC in the CC, and validated candidate regulatory signaling pathways in the pathological process through functional experiments (26). However, the cellular composition, transcriptomic dynamics, and the role of immune cells within the microenvironment in ARED patients remain unclear. A study has shown significant heterogeneity in fibroblasts within rat CC during aging (68). However, differences in CC composition and functional regulation between humans and animal models, such as rats, complicate direct comparisons (14, 15). To address these challenges, we conducted a comprehensive transcriptome profiling to explore the complexity of the CC and its cell type-specific molecular features in human ARED. Due to the challenges in procuring human penile tissue samples from the elderly normal cohort, these samples were not included as controls in this study. This limitation prevented the comparison of molecular changes between the elderly ED and the age-matched normal group. Nevertheless, comparative analyses against the young normal cohort still provide valuable insights for characterizing the cellular landscape of ARED. By employing single-cell analysis, we created a transcriptome atlas of the CC in the context of ARED, enhancing our understanding of its cellular composition and pathogenesis.

We first analyzed the cellular composition of the human CC and identified nine distinct cell subsets. FB, EC, SMC, and pericytes (PC) constituted the major cellular components, consistent with previous study, and have been shown to play crucial roles in both normal erectile function and ED (66, 69). Despite some inter-sample heterogeneity and a loss of cellular identity (particularly in EC), the overall cellular composition remained relatively stable with aging. With advancing age and ED progression, we noted a decrease in the proportion of EC and an increase in immune cells.

Our data indicate that ARED significantly affects stromal cells, potentially leading to structural and functional damage in the penile CC. Compared to immune cells, stromal cells exhibited more DEGs and weaker cellular interactions in ARED. Pairwise differential expression analysis across cell types revealed characteristic features of ARED, including increased endoplasmic reticulum stress (specifically related to the unfolded protein response), heightened inflammation, and elevated oxidative stress and apoptosis. The gene *HSPA5*, an endoplasmic reticulum chaperone crucial for protein folding and quality control, showed progressively higher expression with aging and ED progression, although future studies with larger sample size are required to confirm this trend. Furthermore, we observed reductions in actin filament-based processes, cell junction organization, and the cGMP-PKG signaling pathway, which is vital for vasorelaxation. Importantly, most transcriptional changes were evident in the intermediate stage of ED.

The balance between contraction and relaxation of cavernous smooth muscle is crucial for penile function. Neurotransmitters (e.g., nitric oxide) and vasoactive substances such as calcitonin gene-related peptide (CGRP), vasoactive intestinal polypeptide (VIP), prostaglandin E1 (PGE1), and adenosine act on SMCs, triggering intracellular signaling cascades mediated by the second messengers cGMP and cAMP, which then decrease intracellular calcium concentrations and promote relaxation (8). In ARED, we observed decreased expression of key relaxation-promoting enzymes: guanylate cyclase (*GUCY1A1*, synthesizes cGMP), cGMP-specific protein kinase (*PRKG1)*, adenylate cyclase (*ADCY3*/5/9, convert ATP to cAMP) and the catalytic subunit of cAMP-dependent protein kinase (*PRKACB*). Conversely, genes associated with SMC contraction, such as *RHOA*, *MYLK*, *MYL6*, and *MYL9*, showed increased expression in ARED. This imbalance between cavernosal vasorelaxation and vasoconstriction—characterized by reduced smooth muscle relaxation and/or increased contraction—contributes to ED. Reduced *PDE5A* levels and uneven distribution of other classic signaling receptors regulating tone suggest the need for alternative therapeutic targets. Potential strategies include soluble guanylate cyclase (sGC) stimulators and activators, which may elevate sGC levels in the absence of nitric oxide and demonstrate synergistic and additive effects on endogenous NO (70). Additionally, Rho-kinase inhibitors, such as fasudil, have shown promise in enhancing penile erection and could be beneficial for treating male ED (71). Within the PI3K-AKT-FOXO pathway, decreased *FOXO3* expression may affect vascular homeostasis and vascular aging (72), making it a potential therapeutic target.

Our study identified EC as the second most abundant population in the human CC, following FB. These cells exhibited signs of endothelial dysfunction with aging and ED progression. Subcluster analysis of EC revealed the heterogeneity of EC, identified unique transcriptomic characteristics and specific transcription factors (e.g., MAFB, FOXC2) in cavernosal trabecular EC. Notably, we identified a subset of EC with high levels of *PTGS2* expression, which showed increased activity in the hypoxia, NFkB, and TNFa pathways, potentially rendering them more vulnerable to hypoxia and inflammatory responses. This finding underscores the notion that ED may serve as an early indicator of future cardiovascular disease (CVD) events. Metabolic activity varied among EC subclusters and ED groups. The EC1_PTGS2^high^ subcluster exhibited heightened activity in oxidative phosphorylation and glycolysis, reflecting their increased energy demands for maintaining function. In ARED, inflammation and EC apoptosis emerged as key pathological changes, particularly during mild ED stages, resembling patterns observed in diabetic ED (26). Furthermore, endothelial injury can lead to impaired endothelium-dependent vasodilation.

In the penile CC, immune cells represent a crucial cell population. Our scRNA-seq revealed significant heterogeneity among these immune cells. Notably, we identified a subset of NK cells (NK_CX3CR1, expressing cytotoxic marker) whose functional decline may contribute to the increased susceptibility to ED in the elderly (73). Cell-cell interaction analysis suggested that ligands such as HLA-E from other cell types may influence the immunosuppressive receptors on NK cells, potentially reducing their cytotoxicity. We further identified macrophage subclusters with pro- and anti-inflammatory characteristics within the immune microenvironment. The pro-inflammatory Macro_CXCL8 subcluster was activated in ARED, contributing to an intensified inflammatory immune response. Furthermore, CellChat analysis revealed enhanced ligand-receptor pairs such as CXCL2/3/8(Macro)–ACKR1(stromal cells) and HLA-E (Macro)–KLRK1(NK) in ARED, which may influence the penile microenvironment and contribute to the disease progression.

Based on the above findings, we propose a potential model elucidating the pathogenesis of ARED (**Figure 6H**), which primarily involves two interconnected core processes: stromal cell functional decline and the formation of an inflammatory microenvironment. EC, serving as sensitive sensors, exhibit function dysfunction in response to stressors such as oxidative stress and hypoxia. The apoptosis of SMC and the disruption of contractile-relaxation balance (upregulated contraction-related genes), collectively undermining the structural and functional integrity of the CC. Dysregulated stromal cells (mainly EC) actively recruit and activate immune cells through the release of chemokines (e.g., CXCL2). Activated macrophages further amplify inflammatory signaling by secreting factors such as CXCL2/3/8 and IL1B, establishing an inflammatory microenvironment. This sustained inflammatory signaling and stressful conditions, in turn, exacerbate stromal cell dysfunction (e.g., fibrosis), collectively driving structural remodeling and functional decompensation of the CC, promoting the progression of ED. Notably, our data indicate that these key transitions are largely completed by the moderate ED stage, providing a critical window for early clinical intervention.

The study has several limitations that should be acknowledged. First, regarding the experimental design, the small sample size (n = 2) in the OsED group may affect the robustness of the statistical analysis results. Second, our conclusions are primarily based on bioinformatic analyses and lack validation through functional experiments, such as gene knockdown or overexpression. Consequently, the current evidence can only suggest correlations between the identified molecules and the phenotype, not a causal relationship, and the underlying key molecular mechanisms remain to be further verified.

In summary, we present a detailed analysis of the cellular composition in ARED and offer new insights into how gene expression patterns are altered, particularly in SMC, EC, and immune cells. Our findings lay the groundwork for exploring the cellular and molecular underpinnings of ARED, paving the way for advancements in clinical treatment strategies for this condition.

## Data Availability

The datasets presented in this study can be found in online repositories. Specifically, the raw sequence data generated in this paper have been deposited in the Genome Sequence Archive (Genomics, Proteomics & Bioinformatics 2021) in National Genomics Data Center (Nucleic Acids Res 2022), China National Center for Bioinformation / Beijing Institute of Genomics, Chinese Academy of Sciences, under the accession number GSA-Human: HRA010374 (74, 75).
