# Supplementary material for: Single-cell atlas of human penile corpus cavernosum reveals cellular and functional heterogeneity of aging-related erectile dysfunction
Source: Front Endocrinol (Lausanne). 2025 Oct 29;16:1671482. doi: 10.3389/fendo.2025.1671482 (PMC12605210; doi:10.3389/fendo.2025.1671482)
Supplement: Supplementary file 1 [file Image1.pdf]

A

(Zhao et al., 2022)

Group	Erectile Function	Sample ID	Age(Year)	Estimated Number of Cells	Cell Number (after QC)
Younger	Normal	YNormal_1_Zhao	52	9,338	8,449
Younger	Normal	YNormal_2_Zhao	55	6,558	5,869
Younger	Normal	YNormal_3_Zhao	46	15,263	12,750
Older	Mild ED	OmED_1	66	4,554	4,024
Older	Mild ED	OmED_2	67	42,906	16,031
Older	Mild ED	OmED_3	68	22,271	16,121
Older	Mild ED	OmED_4	72	20,634	8,852
Older	Severe ED	OsED_1	74	4,547	1,933
Older	Severe ED	OsED_2	91	17,201	9,714

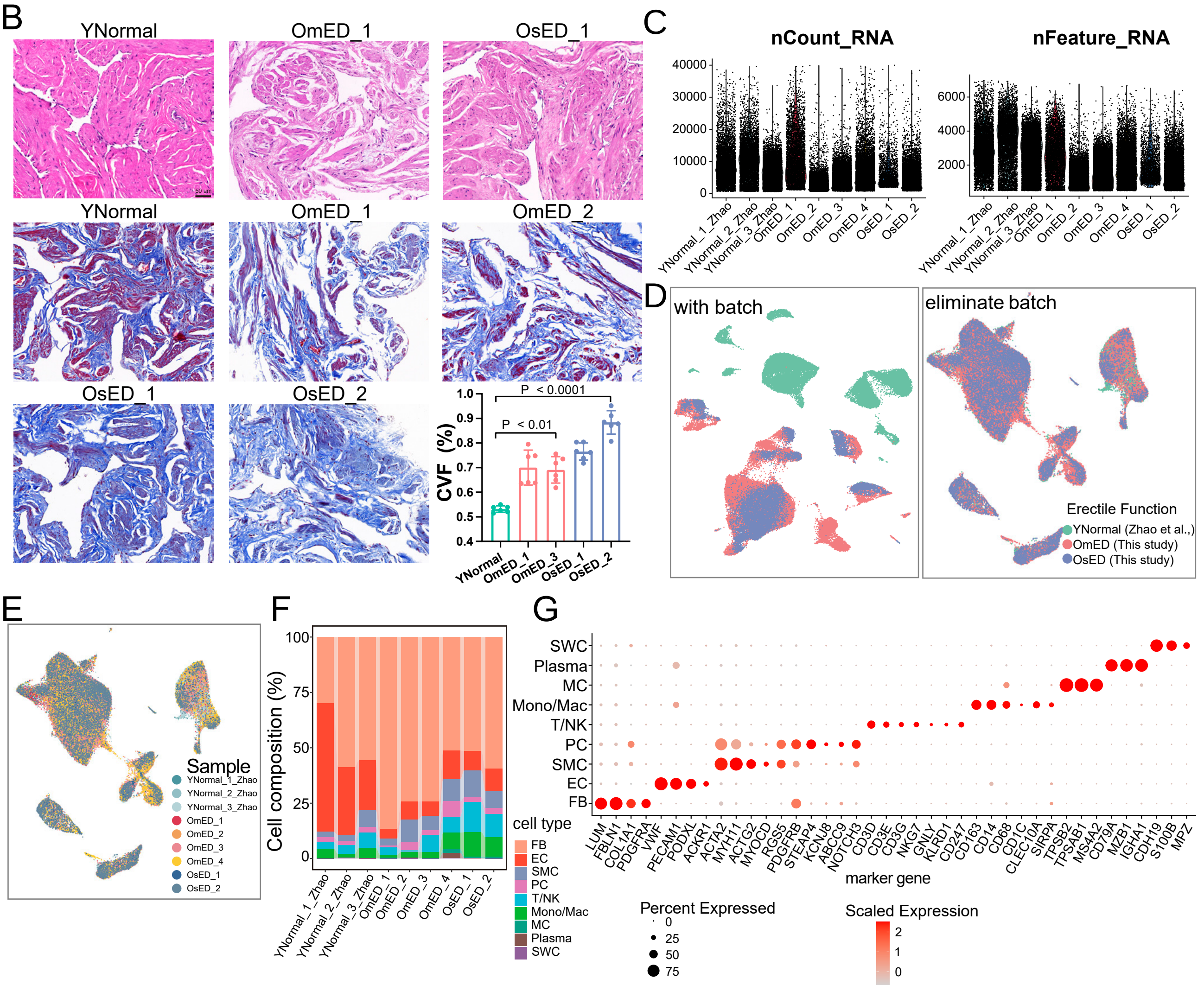

Figure S1. Information on the human penile samples and quality control of scRNA-seq data. **(A)** Collected Clinical information and cell numbers of nine samples. **(B)** H&E and Masson's Trichrome Stain (scale bar = 50 um). Bar plots showing the collagen volume fraction (CVF) in three groups of samples. Note that CVF was significantly increased in OmED and OsED compared to YNormal. **(C)** Violin plots showing the distribution of expressed gene number, UMI number in each sample. **(D)** UMAP plots showing the cell distribution before (left) and after (right) the removal of batch effects. Cells are colored by three groups of erectile function. **(E)** The UMAP plot showing the cell distribution for each sample. Cells are colored by samples. **(F)** Cell proportion of each cell type across nine individuals. **(G)** Bubble diagram of canonical marker genes across nine major cell types. Grey indicates low expression and red indicates high expression.
