# Supplementary material for: Single-cell atlas of human penile corpus cavernosum reveals cellular and functional heterogeneity of aging-related erectile dysfunction
Source: Front Endocrinol (Lausanne). 2025 Oct 29;16:1671482. doi: 10.3389/fendo.2025.1671482 (PMC12605210; doi:10.3389/fendo.2025.1671482)
Supplement: Supplementary file 2 [file Image2.pdf]

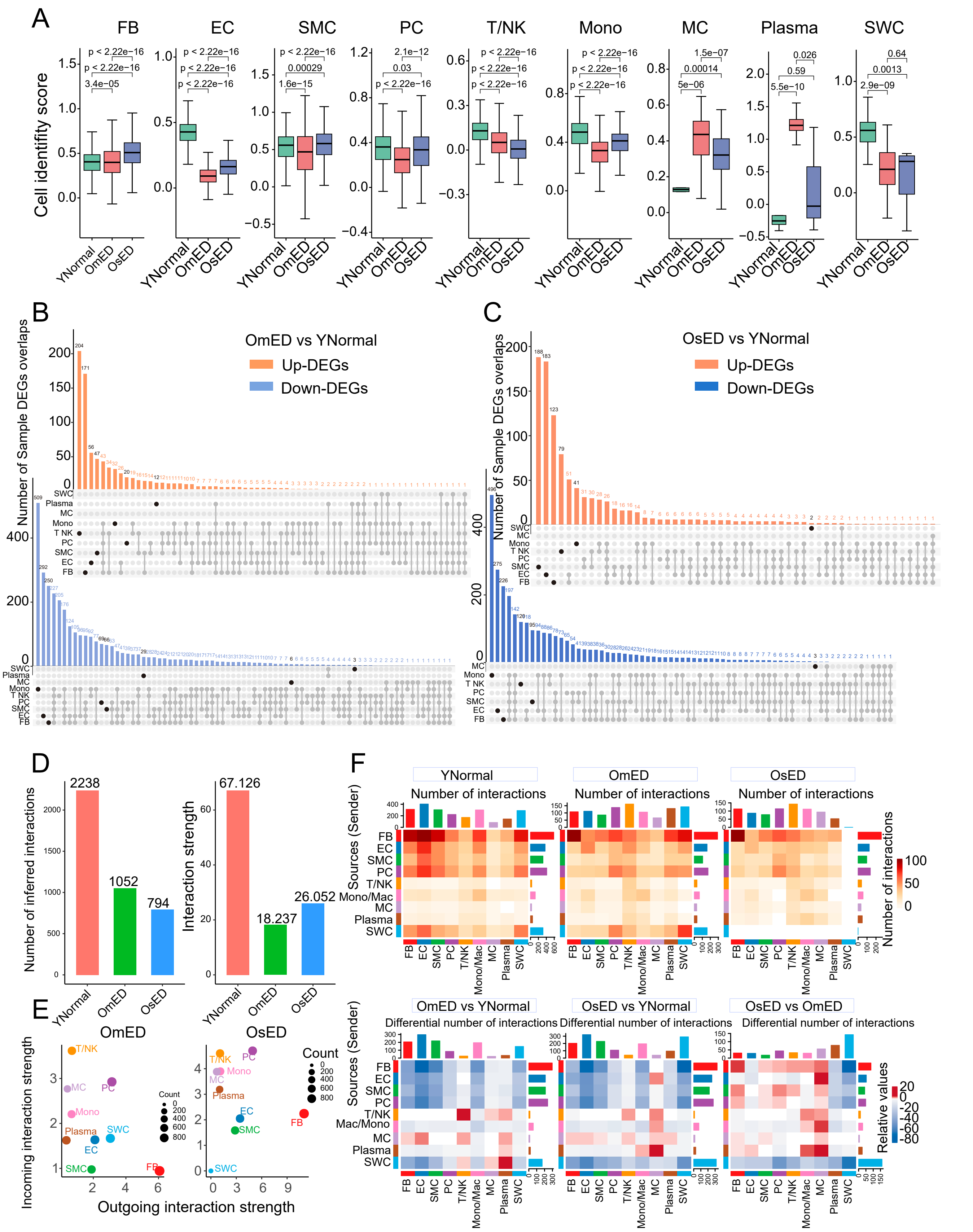

Figure S2. Cell identity analysis and cell-cell communication network of different cell types.
(A) Box plot showing the cell identity score of each cell type in different groups. The P values by Wilcoxon test are indicated in the graphs. **(B-C)** Upset plots displaying the numbers of cell-type-specific and shared up-regulated/down-regulated DEGs in the comparisons between OmED and YNormal (B) as well as between OsED and YNormal (C). **(D)** The barplot showing the total inferred interactions and interaction strength in different groups. **(E)** Scatter plots in a 2D space identifying the cell types with significant changes in sending or receiving signals between different groups. **(F)** Heatmap showing the cell-cell interaction numbers across nine cell types in different groups (top) and differential number of interactions among different cell types across three groups. Red represents increased, while blue represents decreased.
