# Supplementary material for: Single-cell atlas of human penile corpus cavernosum reveals cellular and functional heterogeneity of aging-related erectile dysfunction
Source: Front Endocrinol (Lausanne). 2025 Oct 29;16:1671482. doi: 10.3389/fendo.2025.1671482 (PMC12605210; doi:10.3389/fendo.2025.1671482)
Supplement: Supplementary file 3 [file Image3.pdf]

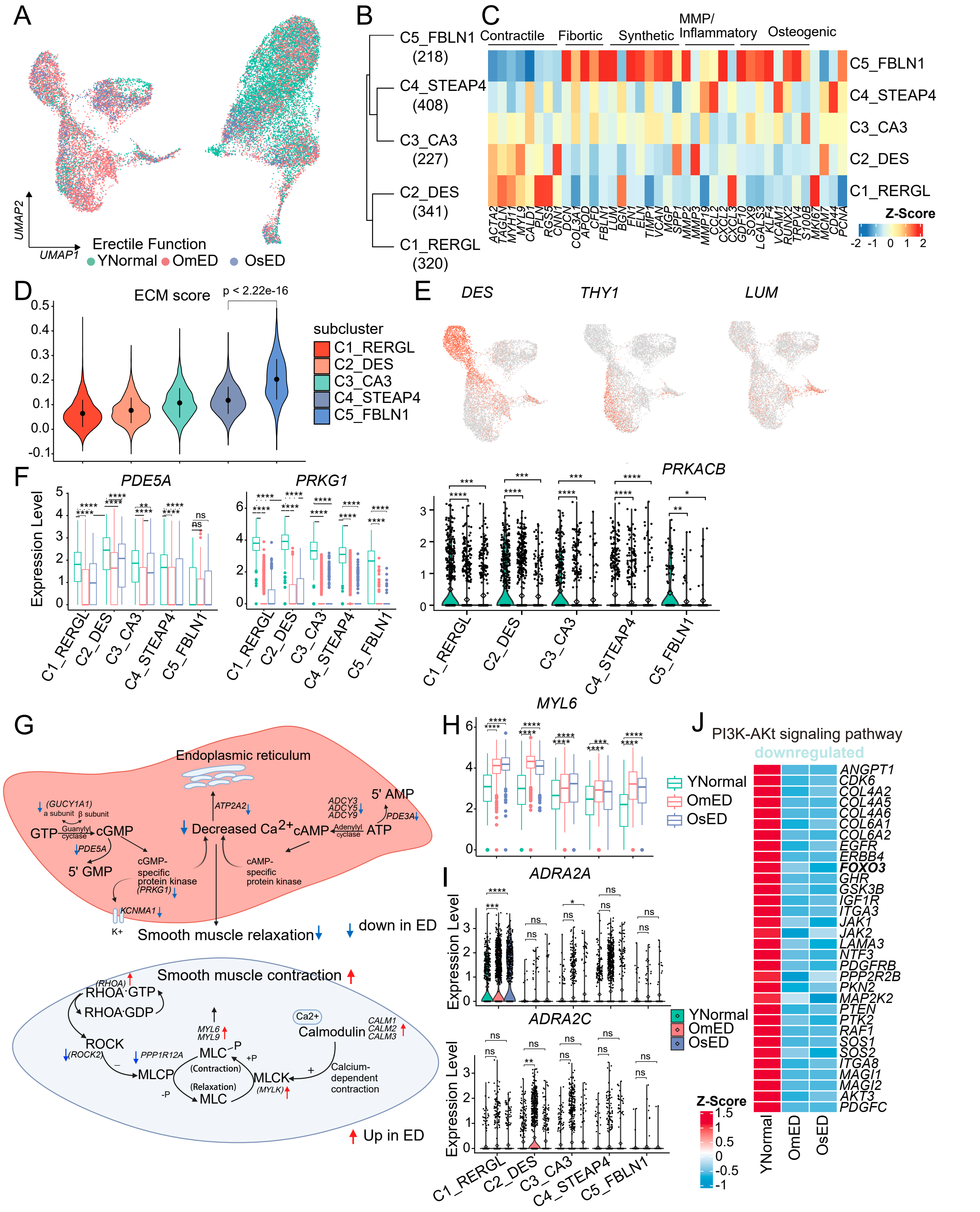

Figure S3. Transcriptional heterogeneity of SMC subclusters and between YNormal and ARED.
(A) UMAP plot showing the distribution of three erectile function groups. **(B)** Clustering analysis of DEGs showing the relationships of subclusters. The value represents the number of DEGs. **(C)** Heatmap showing the expression levels of genes that associated with different functions in the five subclusters. **(D)** ECM components score for each subcluster shown by violin plots. **(E)** UMAP plots showing the expression pattern of selected marker genes in the SMC and PC clusters. **(F)** Box plots and violin plots showing the expression of *PDE5A* ,*PRKG1 and PRKACB* in different SMC subclusters between YNormal and ARED groups. Box plots indicate the median, the quartile range (25%-75%) and outliers (single points). ns, not significant; *, p.adj < 0.05; **, p.adj < 0.01; ***, p.adj < 0.001; ****, p.adj < 0.0001 (two-sided Wilcoxon rank-sum test). **(G)** A schematic showing core upregulated and downregulated components in the cGMP-PKG signaling pathway. **(H-I)** The expression levels of *MYL6* (H), *ADRA2A* and *ADRA2C* (I) in five subclusters of different groups. ns, not significant; *, p.adj < 0.05;**, p.adj < 0.01; ***, p.adj < 0.001; ****, p.adj < 0.0001 (two-sided Wilcoxon rank-sum test). **(J)** Heatmap showing the relative expression levels of downregulated genes related to PI3K-Akt signaling pathway in C1_RERGL across different groups.
