# Supplementary material for: Single-cell atlas of human penile corpus cavernosum reveals cellular and functional heterogeneity of aging-related erectile dysfunction
Source: Front Endocrinol (Lausanne). 2025 Oct 29;16:1671482. doi: 10.3389/fendo.2025.1671482 (PMC12605210; doi:10.3389/fendo.2025.1671482)
Supplement: Supplementary file 4 [file Image4.pdf]

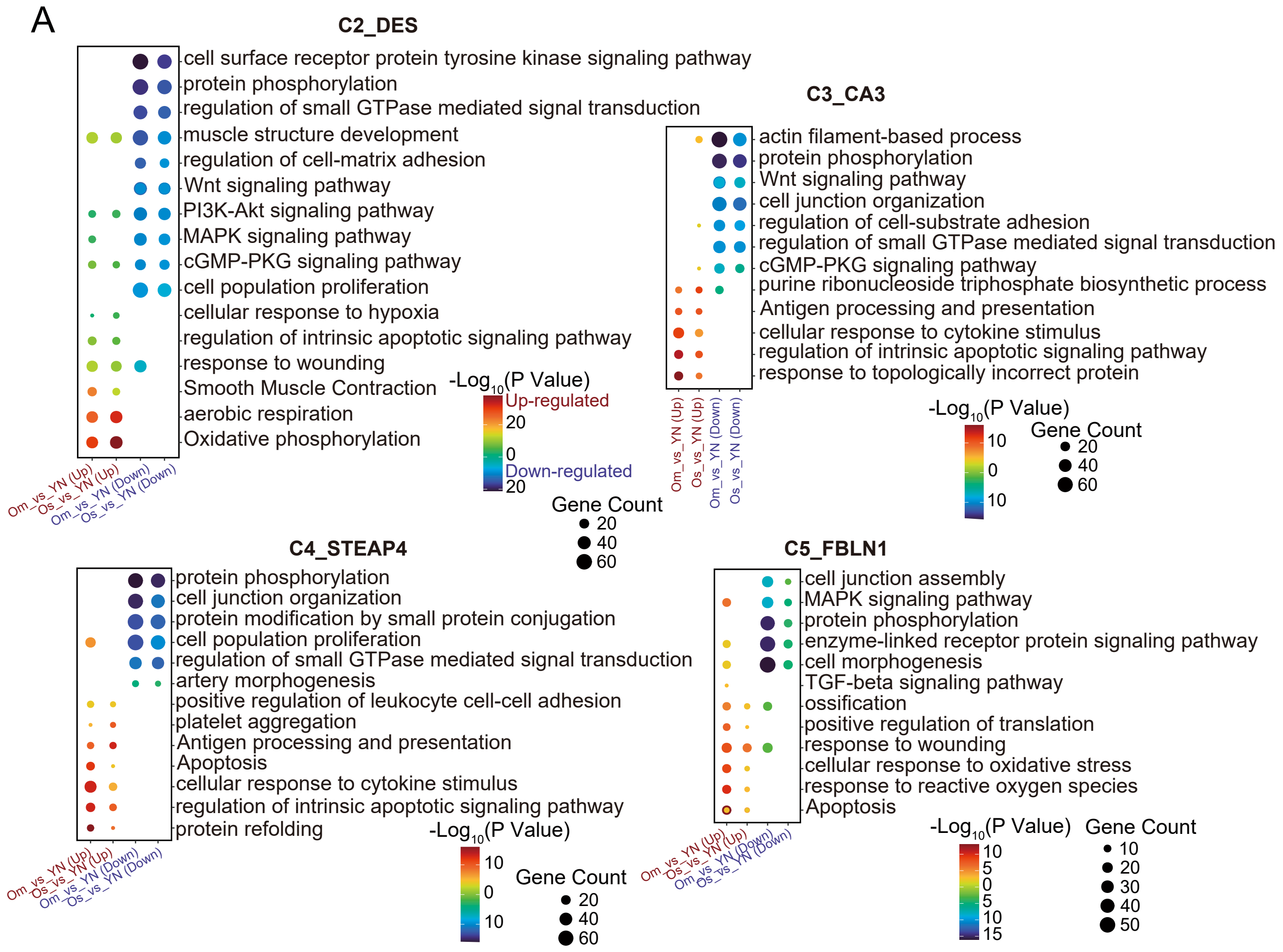

Figure S4. Pathway analysis of DEGs between YNormal and ARED for four SMC subclusters. **(A)** Dot plots showing the representative shared pathways of upregulated (red color) and downregulated (blue color) DEGs across each subclusters for comparison between OmED/OsED and YNormal.

Abbreviations: Om_vs_YN: OmED vs YNormal; Os_vs_YN: OsED vs YNormal.
