# Supplementary material for: Single-cell atlas of human penile corpus cavernosum reveals cellular and functional heterogeneity of aging-related erectile dysfunction
Source: Front Endocrinol (Lausanne). 2025 Oct 29;16:1671482. doi: 10.3389/fendo.2025.1671482 (PMC12605210; doi:10.3389/fendo.2025.1671482)
Supplement: Supplementary file 5 [file Image5.pdf]

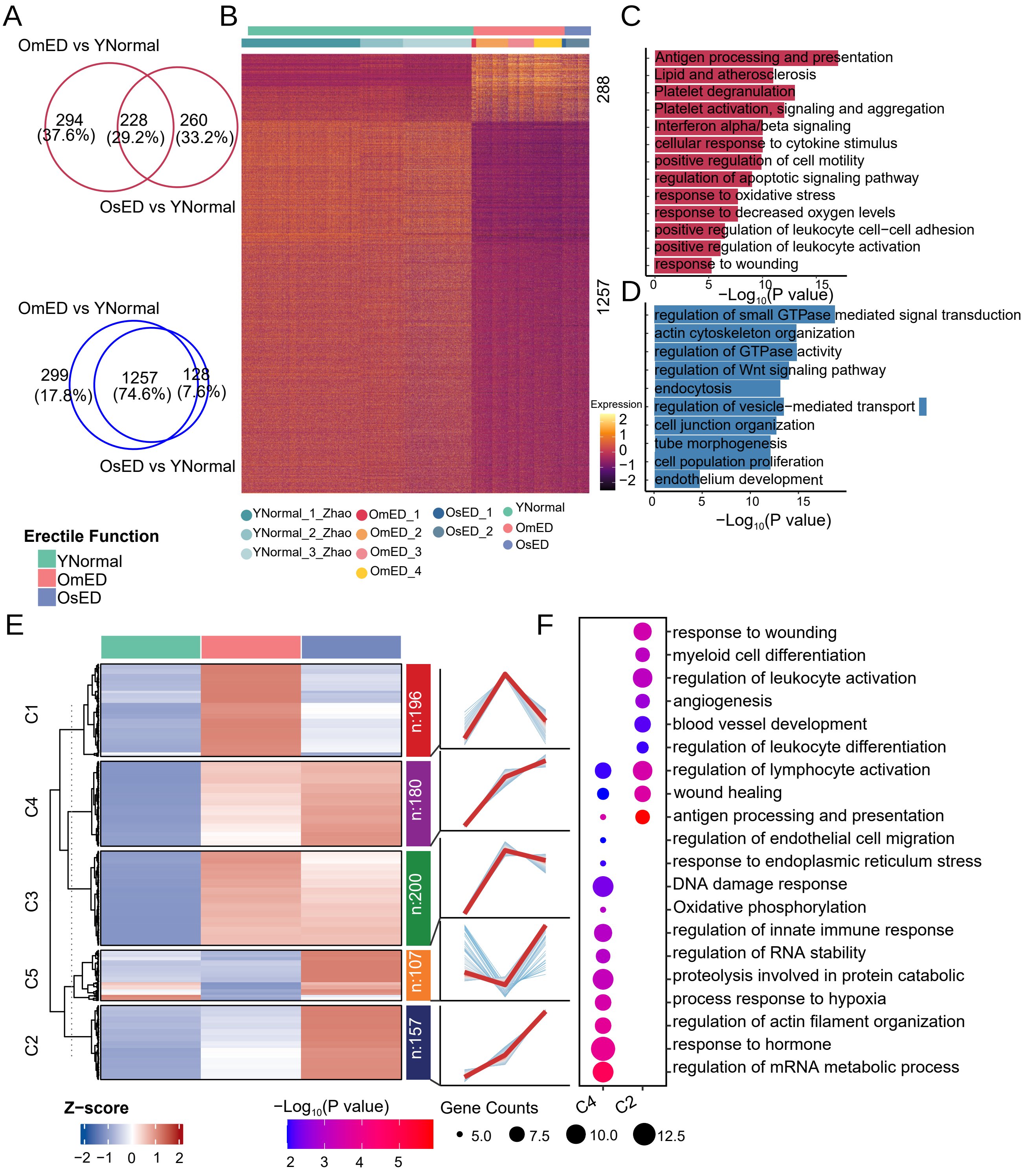

Figure S5. Endothelial dysfunction in ARED.
(A) Venn plots showing the overlaps of upregulated (top) and downregulated (bottom) genes in OmED and OsED EC (compared with YNormal). **(B)** The expression signatures of shared upregulated and downregulated genes in (A). **(C-D)** Bar plots showing the representative pathways of shared upregulated genes (C) or downregulated genes (D) in OmED and OsED. **(E)** Heatmap and line plots showing five gene clusters obtained via the soft clustering method (Mfuzz) in merged upregulated DEGs after pairwise comparisons. **(F)** Representative pathways of C2 and C4 gene clusters.
