# Supplementary material for: Single-cell atlas of human penile corpus cavernosum reveals cellular and functional heterogeneity of aging-related erectile dysfunction
Source: Front Endocrinol (Lausanne). 2025 Oct 29;16:1671482. doi: 10.3389/fendo.2025.1671482 (PMC12605210; doi:10.3389/fendo.2025.1671482)
Supplement: Supplementary file 6 [file Image6.pdf]

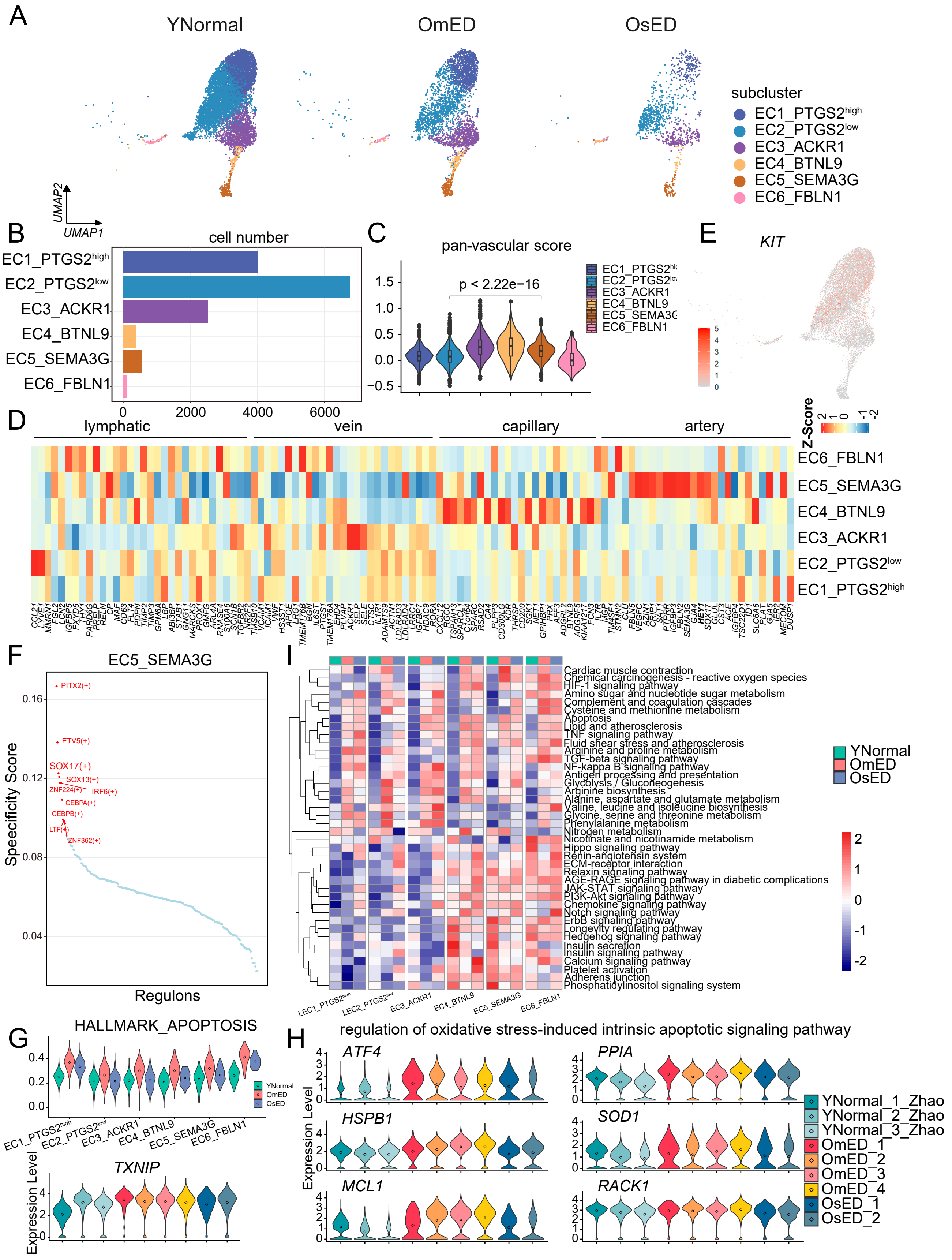

Figure S6. Heterogeneity of EC subclusters and transcriptional changes in ARED. **(A)** UMAP plots showing the distributions of six EC subclusters divided by YNormal, OmED and OsED groups. **(B)** Bar plot showing the numbers of six EC subclusters. **(C)** Violin plot and box plot showing the pan-vascular associated gene set scores in six EC subclusters. **(D)** Heatmap showing the expression pattern of genes associated with endothelial phenotypes in six EC subclusters. **(E)** UMAP plot of the expression distribution of *KIT*, a marker gene in cavernosal trabecular EC. **(F)** Dot plot showing the TF regulons in EC5_SEMA3G. The top 10 TFs are highlighted. **(G)** Violin plots showing the "HALLMARK_ APOPTOSIS" gene set score between different groups in different subclusters of EC (top) and the expression level of *TXNIP* in different individuals (bottom). **(H)** Violin plots showing the expression levels of "regulation of oxidative stress-induced intrinsic apoptotic signaling pathway" related genes upregulated in OmED for EC5_SEMA3G subcluster. **(I)** Heatmap showing different expression patterns of KEGG pathways among different groups in six EC subclusters calculated by gene set variation analysis (GSVA).
