# Supplementary material for: Single-cell atlas of human penile corpus cavernosum reveals cellular and functional heterogeneity of aging-related erectile dysfunction
Source: Front Endocrinol (Lausanne). 2025 Oct 29;16:1671482. doi: 10.3389/fendo.2025.1671482 (PMC12605210; doi:10.3389/fendo.2025.1671482)
Supplement: Supplementary file 7 [file Image7.pdf]

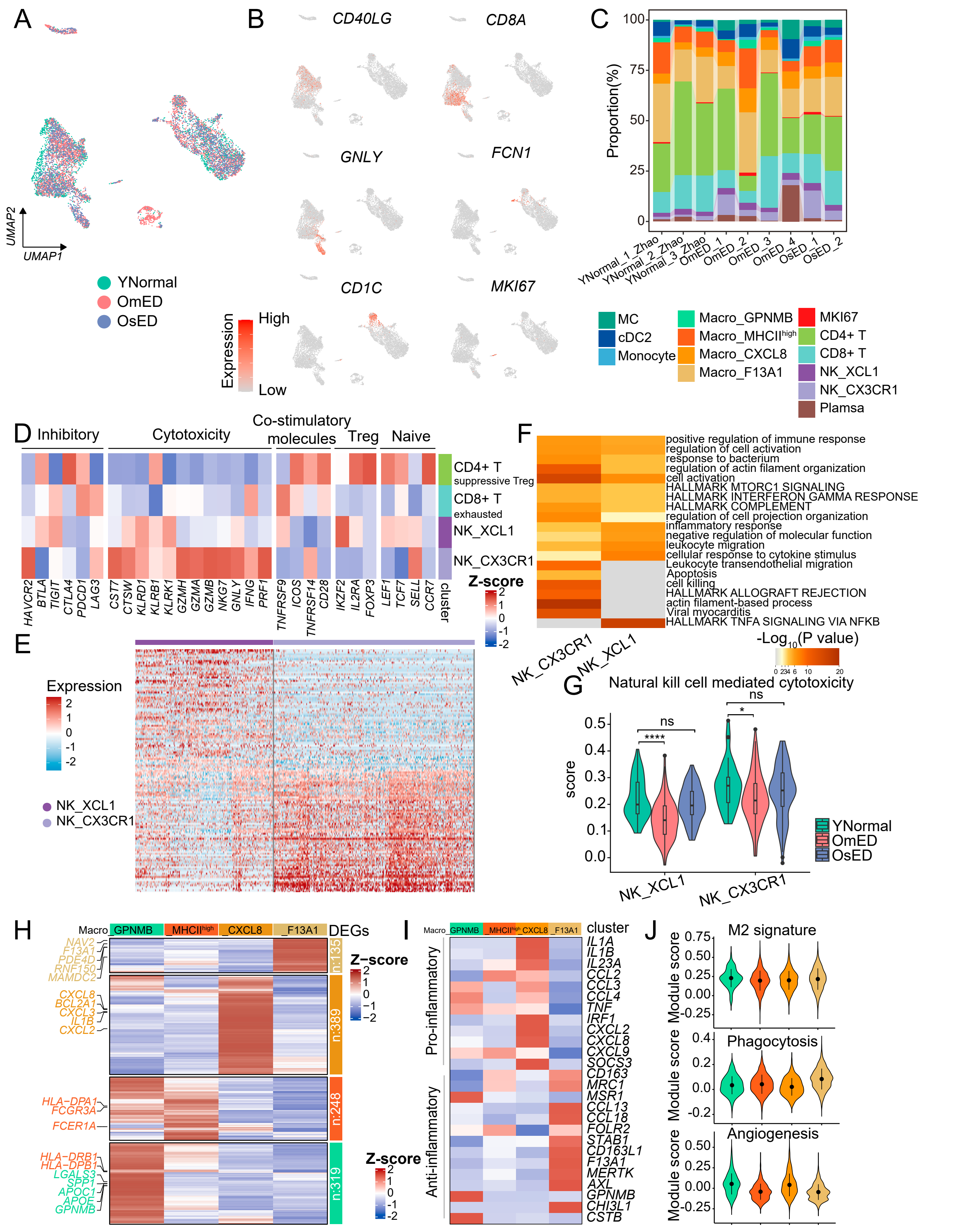

Figure S7. Heterogeneity of lymphoid and myeloid subclusters.
(A) UMAP plot of immune subclusters in YNormal and ARED. **(B)** UMAP plot showing expression of marker genes for some immune subclusters. **(C)** Cell proportion of immune subclusters per sample. **(D)** The expression of cell functions associated genes in CD4+ T, CD8+ T and two NK subclusters. **(E)** Heatmap showing the expression signatures of DEGs in two NK subclusters. **(F)** Heatmap showing representative pathways of DEGs between two NK subclusters. **(G)** Violin and box plot of the Natural kill cell mediated cytotoxicity score of two NK subclusters across YNormal and ARED. Box shows the median and the quartilerange (25%-75%). ns, not significant; *, p.adj < 0.05; ****, p.adj < 0.0001 (two-sided Wilcoxon rank-sum test). **(H)** Heatmap of marker genes of four Macro subclusters. **(I)** Heatmap showing the expression of Pro- and Anti-inflammatory associated genes in four Macro subclusters. **(J)** Geneset score analysis of "M2 signature"," Phagocytosis" and "Angiogenesis" in four Macro subclusters.
