# Supplementary material for: Single-cell atlas of human penile corpus cavernosum reveals cellular and functional heterogeneity of aging-related erectile dysfunction
Source: Front Endocrinol (Lausanne). 2025 Oct 29;16:1671482. doi: 10.3389/fendo.2025.1671482 (PMC12605210; doi:10.3389/fendo.2025.1671482)
Supplement: Supplementary file 8 [file Image8.pdf]

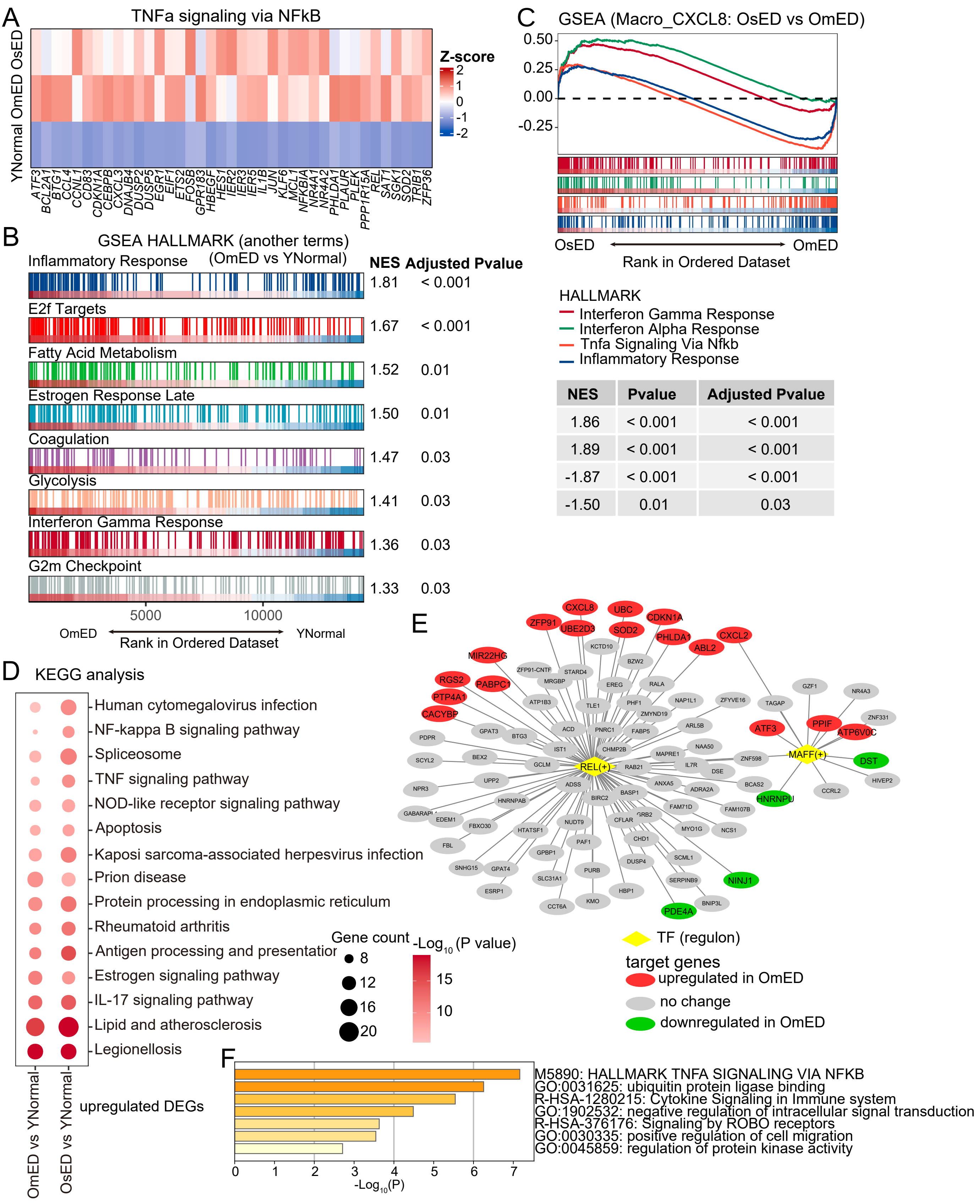

Figure S8. Pathway and TF regulon analysis of Macro_CXCL8 in penile CC microenvironment. **(A)** Heatmap showing the expression of genes related to "TNFa signaling via NFkB" in YNormal, OmED and OsED groups. **(B)** Gene set enrichment analysis (GSEA) showing other significant HALLMARK pathways in OmED Macro_CXCL8 compared to YNormal Macro_CXCL8. Ranked genes are showed. **(C)** Gene set enrichment analysis (GSEA) showing HALLMARK pathways significant changed in OsED Macro_CXCL8 compared to OmED Macro_CXCL8. **(D)** Dot plot showing the shared representative KEGG pathways of upregulated genes in OmED and OsED compared with YNormal in Macro_CXCL8 subcluster. **(E)** The transcriptional regulated network showing the target genes regulated by two TF regulons (highlighted in yellow color). The genes in the red ovals represent upregulated genes in OmED group in Macro_CXCL8 subcluster, while the genes in the green ovals represent downregulated genes. **(F)** The representative pathways for upregulated genes in the (E) diagram.
