# Supplementary material for: Single-cell atlas of human penile corpus cavernosum reveals cellular and functional heterogeneity of aging-related erectile dysfunction
Source: Front Endocrinol (Lausanne). 2025 Oct 29;16:1671482. doi: 10.3389/fendo.2025.1671482 (PMC12605210; doi:10.3389/fendo.2025.1671482)
Supplement: Supplementary file 9 [file Image9.pdf]

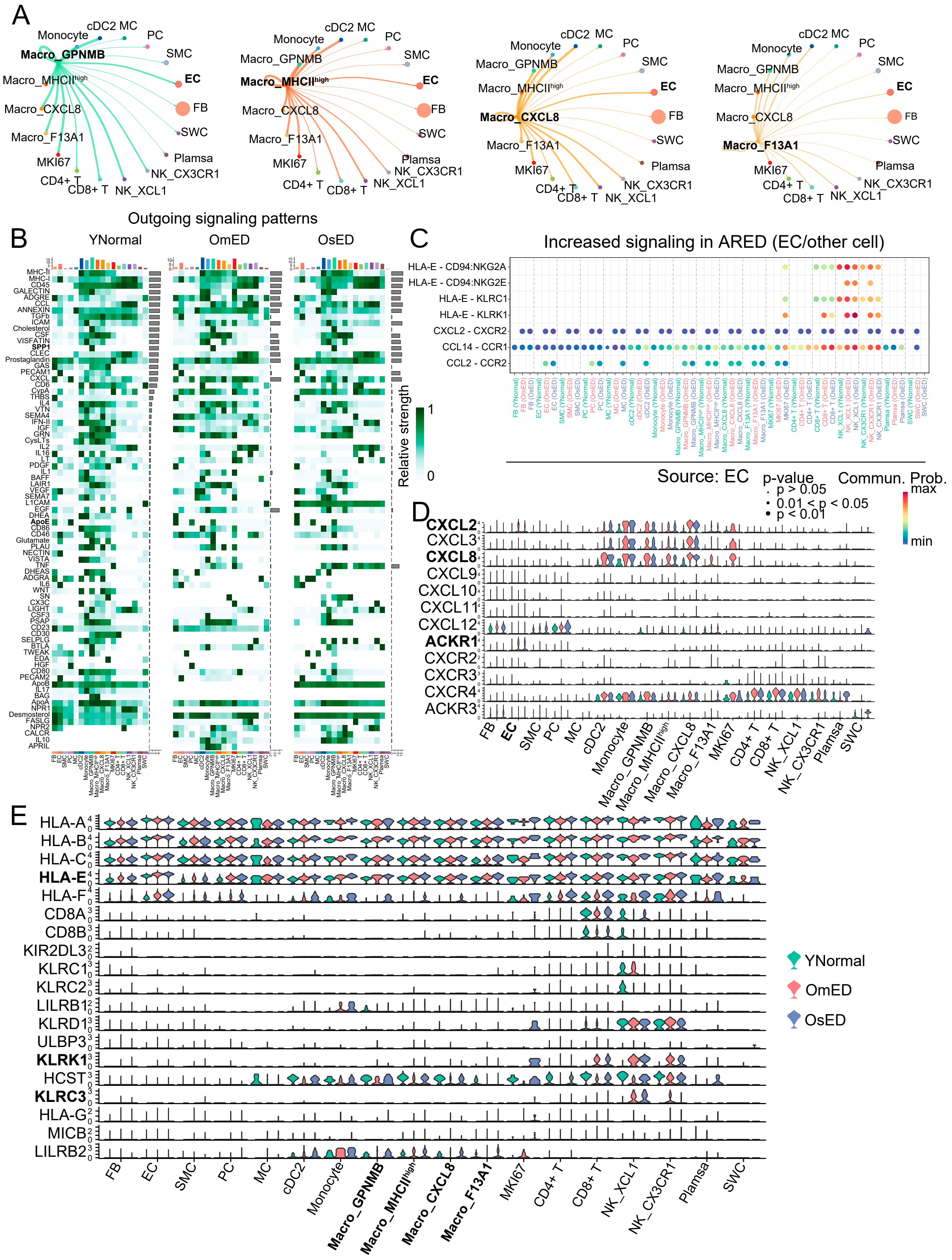

Figure S9. Cell-cell communications between subclusters in human penile CC.
(A) Cell-cell communication network showing the signaling sent from four Macro subclusters. Edge weights are proportional to the interaction strength. The thicker the edge line, the stronger signal. **(B)** Heatmap of the CellChat outgoing signaling in each Macro subclusters. **(C)** Bubble plot showing the up-regulated signaling ligand-receptor pairs in ARED between EC and other cell types. **(D)** Violin plotshowing the CXCL signaling gene expression distribution between YNormal and ARED. **(E)** Violin plotshowing the MHC-I signaling gene expression distribution between YNormal and ARED.
